# Do regional brain volumes and major depressive disorder share genetic architecture? A study of Generation Scotland (*n*=19 762), UK Biobank (*n*=24 048) and the English Longitudinal Study of Ageing (*n*=5766)

**DOI:** 10.1038/tp.2017.148

**Published:** 2017-08-15

**Authors:** E M Wigmore, T-K Clarke, D M Howard, M J Adams, L S Hall, Y Zeng, J Gibson, G Davies, A M Fernandez-Pujals, P A Thomson, C Hayward, B H Smith, L J Hocking, S Padmanabhan, I J Deary, D J Porteous, K K Nicodemus, A M McIntosh

**Affiliations:** 1 Division of Psychiatry, University of Edinburgh, Royal Edinburgh Hospital, Edinburgh, UK; 2 Centre for Cognitive Ageing and Cognitive Epidemiology, University of Edinburgh, Edinburgh, UK; 3 Centre for Genomic and Experimental Medicine, Institute of Genetics and Molecular Medicine, University of Edinburgh, Western General Hospital, Edinburgh, UK; 4 Division of Population Health Sciences, University of Dundee, Dundee, UK; 5 Division of Applied Medicine, University of Aberdeen, Aberdeen, UK; 6 Institute of Cardiovascular and Medical Sciences, University of Glasgow, Glasgow, UK; 7 Department of Psychology, University of Edinburgh, Edinburgh, UK

## Abstract

Major depressive disorder (MDD) is a heritable and highly debilitating condition. It is commonly associated with subcortical volumetric abnormalities, the most replicated of these being reduced hippocampal volume. Using the most recent published data from Enhancing Neuroimaging Genetics through Meta-analysis (ENIGMA) consortium’s genome-wide association study of regional brain volume, we sought to test whether there is shared genetic architecture between seven subcortical brain volumes and intracranial volume (ICV) and MDD. We explored this using linkage disequilibrium score regression, polygenic risk scoring (PRS) techniques, Mendelian randomisation (MR) analysis and BUHMBOX. Utilising summary statistics from ENIGMA and Psychiatric Genomics Consortium, we demonstrated that hippocampal volume was positively genetically correlated with MDD (*r*_G_=0.46, *P*=0.02), although this did not survive multiple comparison testing. None of the other six brain regions studied were genetically correlated and amygdala volume heritability was too low for analysis. Using PRS analysis, no regional volumetric PRS demonstrated a significant association with MDD or recurrent MDD. MR analysis in hippocampal volume and MDD identified no causal association, however, BUHMBOX analysis identified genetic subgrouping in GS:SFHS MDD cases only (*P*=0.00281). In this study, we provide some evidence that hippocampal volume and MDD may share genetic architecture in a subgroup of individuals, albeit the genetic correlation did not survive multiple testing correction and genetic subgroup heterogeneity was not replicated. In contrast, we found no evidence to support a shared genetic architecture between MDD and other regional subcortical volumes or ICV.

## Introduction

Major depressive disorder (MDD) is a debilitating condition that accounts for a large proportion of disease burden world-wide.^1^ It is a complex disorder that is influenced by both genetic and environmental factors with a heritability of ~37% estimated from twin studies.^2^ Two recent genome-wide association studies (GWAS) identified two loci in MDD^3^ and 15 loci in self-reported depression^4^ of genome-wide significance. Nevertheless, the majority of MDD’s heritability is unaccounted for by currently identified variants and the mechanisms leading from gene to clinical phenotype remain elusive.

Reports of lower brain volumes in cross-sectional studies are common in MDD, but small sample sizes have potentially contributed to poorly replicated results. Enhancing Neuro-Imaging Genetics through Meta-Analysis (ENIGMA) completed a large MDD case–control meta-analysis of subcortical volumes (*n*=8927) demonstrating a significant association between MDD and reduced hippocampal volume (Cohen’s *d*=−0.20).^5^ Numerous other studies have also demonstrated a link between hippocampal reduction and MDD, and it is one of the most robustly associated brain regions.^6^ Other brain regions have shown limited and sometimes contradictory evidence for association with MDD. Smaller amygdala volume has been associated with depressive symptoms^7, 8^ and MDD status,^9^ however larger amygdala volume has also been associated with the disorder.^10^ A 2013 meta-analysis concluded that, as well as hippocampus, smaller putamen and thalamus volumes were associated with late life MDD, although fewer studies have examined these regions.^11^ In addition, smaller caudate nucleus volumes have also been associated with MDD in a meta-analysis.^12^ The nucleus accumbens has not been widely associated with MDD status but a smaller volume has been implicated in the lethality of suicidal acts within mood disorder sufferers.^13^ Pallidum volume and intracranial volume (ICV) have not been associated with MDD in any meta-analysis to date, as far as we are aware.

Subcortical structural volumes are known to be influenced by both genetic and environmental factors and have been demonstrated to be moderately to highly heritable ranging from 0.44 to 0.88.^14^ The previously reported lower brain volumes in MDD and the relatively high heritability of these structures means they could be of interest as an intermediate phenotype.^14^ Overlap between genes involved in MDD and subcortical regions have been explored previously. The majority of studies have focused on candidate genes, such as the serotonin transporter (5-HTTLPR), and findings have often been contradictory.^15^ As the success of a candidate gene study is reliant on the correct gene being chosen, GWAS studies are often considered to be a less biased and more reliable approach.^16^ GWAS of regional brain volumes has recently been completed by the ENIGMA Consortium,^17^ providing an important opportunity to examine the genetic overlap between subcortical brain volumes and ICV with MDD. Indications of covariation could potentially identify the risk conferring loci involved in MDD as well as the underlying mechanisms.

In this current study, we examine whether the genetic architecture of MDD is shared with multiple subcortical brain regions and ICV. We employed four techniques; the first, linkage disequilibrium (LD) score regression,^18, 19^ estimates the genetic correlation between these traits using GWAS summary statistics from the ENIGMA consortium and Psychiatric Genomics Consortium (PGC). The second method, polygenic risk scoring (PRS),^20^ utilises ENIGMA summary statistics to generate individual level polygenic profile scores of each brain region’s volume. We then calculated the association of PRS (a) with their own volume in UK Biobank and (b) with MDD status in three population-based cohorts; Generation Scotland: Scottish Family Health Study (GS:SFHS), English Longitudinal Study of Ageing (ELSA) and UK Biobank and (c) with recurrent MDD, MDD episodes, MDD duration and age of onset in GS:SFHS and UK Biobank. Both (b) and (c) analyses were adjusted for confounds on an individual subject level and then combined in a meta-analysis. Third, we used the Mendelian randomisation method^21^ to examine a directional causal relationship between the regional volumes and MDD, utilising the GWAS significant loci as genetic instruments. Lastly, we used a new software package BUHMBOX^22^ to test for the presence of genetic subgroup heterogeneity.

## Materials and methods

### Cohort descriptions and genotyping

#### Generation Scotland: Scottish Family Health Study

GS:SFHS is a family-based cohort with phenotypic data for 24 080 participants (mean age=47.6, s.d.=15.4) of which 20 032 had genotype data. Individuals were eligible if they had one first-degree relative willing to partake in the study. Further details on the recruitment for this cohort are available in the Supplementary Materials and have been described previously.^23^ Diagnosis of MDD was made using the structured clinical interview for DSM-IV disorders (SCID) for those individuals that screened positive during interview questions (*n*=19 762, cases=2643).^24^ Individuals with bipolar disorder (*n*=76) were excluded from this study. Information on MDD episodes and age of onset was also included in the SCID and therefore recurrent MDD and duration of MDD could be inferred (further details are given in the Supplementary Materials).

Details of the DNA extraction for GS:SFHS have been previously described.^25^ Genotyping was completed at the Wellcome Trust Clinical Research Facility Genetics Core, Edinburgh (www.wtcrf.ed.ac.uk) using the Illumina HumanOmniExpressExome -8v1.0 Beadchip (San Diego, CA, USA) and Infinium chemistry^26^ and processed using GenomeStudio Analysis Software v2011.1. Quality Control (QC) utilised the following inclusion thresholds; missingness per individual <1%, missingness per single-nucleotide polymorphism (SNP) <1%, Hardy–Weinberg Equilibrium (HWE) *P*-value >1 × 10^−6^, minor allele frequency (MAF) >1%. There were 556 705 SNPs and 19 994 individuals that passed QC criteria.

#### UK Biobank

UK Biobank is an open resource cohort with phenotypic data for 502 664 (mean age=56.5, s.d.=8.1) between the ages of 40–69 recruited within the United Kingdom between 2006 and 2010, with genotype data available for 152 734 participants. Our study was conducted under UK Biobank application 4844. Study design and recruitment has been described previously^27^ but, in brief, participants were asked to complete a touchscreen questionnaire and additional data were collected by nurse interview. MDD status was based upon putative MDD phenotype defined by Smith *et al.*^28^ (*n*=24 048). Participants with mild depressive symptoms were removed based on this definition and self-reported bipolar disorder participants (*n*=1211) were excluded. Information on MDD episodes and age of onset was also available, therefore recurrent MDD and MDD duration was inferred (further details are given in the Supplementary Materials). Subcortical volumes for nucleus accumbens, amygdala, caudate nucleus, hippocampus, pallidum, putamen and thalamus were measured by T1-weighted structural imaging. The UK Biobank imaging protocol has been described elsewhere (http://biobank.ctsu.ox.ac.uk/crystal/refer.cgi?id=1977). The mean of the sum of left and right volume was taken for each subcortical region. ICV was generated by the sum of white matter, grey matter and ventricular cerebrospinal fluid volumes. Imaging data for the eight structures were available for 4446 participants, of which 968 had genetic data available.

Genotyping was completed utilising two Affymetrix arrays (Santa Clara, CA, USA); BiLEVE (*n*=49 979) and the UK Biobank Axiom (*n*=102 750). Details have been described previously.^29^ Initial genotyping QC was performed by UK Biobank.^30^ Additional filtering was then applied to participants with poor heterozygosity or missingness, QC failure, non-British White ancestry, gender mismatch, genotype missingness <2%, and relatedness within UK Biobank and to the GS:SFHS sample (*r*>0.0442, *n*=35 752) and ELSA sample (in the meta-analysis with all three cohorts). SNPs inclusion criteria were HWE *P*>1 × 10^−6^ and MAF>1%. There were 731 536 SNPs and 152 735 individuals that passed QC criteria.

#### English Longitudinal Study of Ageing

ELSA is a prospective cohort study of health and ageing collected in 2002 with six follow-up waves taken at 2-year intervals. At wave 1 (baseline), phenotypic data were available for 12 003 (mean age=63.9, s.d.=10.7) and genotypic data available for 7452 participants. Details of this cohort have been described previously^31^ and further information is available in the Supplementary Materials. MDD status in this study was defined using a shortened form of the Centre of Epidemiological Studies—Depression scale (CES-D scale) (completed by 5752 participants with genomic data). This consisted of 8 questions, rather than the original 20, with a ‘no’/‘yes’ response that was converted to a binary 0/1, respectively, although positive questions, that is, ‘During the past week, were you happy?’, were scored in reverse; 0 being ‘yes’ and 1 being ‘no’. After summing the scores, a dummy variable of MDD status was classified as those with a score of 4 or above, as in previous studies.^32^ Self-reported ‘manic depressive’ (*n*=41) individuals were excluded.

Genotyping was completed in 2013/14 on 7452 participants on the Illumina Omni 2.5–8 chip and QC and removal of related individuals (*r*≥0.2, *n*=109) was completed at the University College London Genetics Institute. Further QC was implemented using the same inclusion thresholds as used for GS:SFHS; SNP inclusion criteria were HWE *P*>1 × 10^−6^ and MAF>1% and exclusion of related individuals (*r*>=0.2, *n*=109). There were >1.3 million SNPs and 7230 individuals that passed QC criteria.

### LD score regression

Genetic correlation of subcortical structures and ICV with MDD were measured using the LD score regression technique.^18, 19^ In brief, this technique utilises GWAS summary statistics to estimate the SNP-based heritability of a trait and genetic correlation between traits, in this study we used summary data from ENIGMA and PGC. SNPs inclusion criteria were INFO>0.9 and MAF>%1 (further details in Supplementary Materials).

Summary statistics for the regional brain volume GWAS completed by ENIGMA were downloaded from http://enigma.ini.usc.edu/research/download-enigma-gwas-results/. The GWAS was completed using 11 840 participants for eight MRI volumetric measures; nucleus accumbens, amygdala, caudate nucleus, hippocampus, pallidum, putamen, thalamus and ICV.^17^

Summary statistics for the MDD GWAS completed by the MDD Working Group of the PGC were downloaded from http://www.med.unc.edu/pgc/downloads. The study examined 9238 MDD cases and 8039 controls.^33^

### Polygenic risk scoring

Construction of PRS was completed in PLINK software.^34^ PRS utilise effect sizes from GWAS summary statistics to construct an additive individual genetic scores in a population.^20^ Summary statistics were taken from the ENIGMA GWAS^17^ (details above) to construct weighted PRS using five *P-*value thresholds: 0.01, 0.05, 0.1, 0.5 and 1, after SNPs underwent clumped-based pruning (*r*^2^=0.25, 300 kb window). All five thresholds are reported in models of subcortical volume and ICV PRS predicting their respective volume in UK Biobank and the best predictive threshold was carried forward into models associating MDD status with each subcortical volume and ICV in all three cohorts. The *P*-value thresholds carried forward were; nucleus accumbens: 0.01, amygdala: 0.1, caudate nucleus: 0.5, hippocampus: 0.01, ICV: 0.5, pallidum: 0.5, putamen: 0.1 and thalamus: 0.05. Scores for GS:SFHS, UK Biobank and ELSA were computed on the raw genotypes.

### Statistical analysis

#### Association between regional brain volume PRS and its respective volume

Models predicting regional brain volumes in UK Biobank were conducted using linear regression in R version 3.2.3 (www.r-project.org). Models were adjusted for age, sex and the first 15 principal components (PCs) as well as for ICV (excepting ICV itself).

#### Association between regional brain volume PRS and MDD

Mixed linear model analyses were completed in ASReml-R (http://www.vsni.co.uk/software/asreml/) for GS:SFHS with MDD status. Mixed linear modelling was utilised to account for the family structure in GS:SFHS. MDD status was fitted as the dependent variable and volume PRS fitted as the independent variable. The model was adjusted for age and sex with the first four PCs fitted to control for population stratification. An additive relationship matrix (expected relatedness derived from pedigree information) was fitted as a random effect to account for the family structure in GS:SFHS. Wald’s conditional F-test was used to calculate *P*-values for all fixed effects and the variance explained was calculated by division of the difference in the sum of residual variance and additive genetic effect in the null model (without PRS) with the full model (with PRS). To adjust for the use of linear-mixed regression models being applied to a binary dependent variable in a structured data set, the fixed effects and standard errors from the linear model were transformed utilising a Taylor series approximation^35^ from the linear scale to the liability scale (Supplementary Materials).

In unrelated samples (UK Biobank and ELSA) logistic regression utilising generalised linear models in R version 3.2.3 (www.r-project.org) was used to test the degree of association between MDD and PRS of subcortical volumes and ICV. Models were adjusted for age, sex and the first 15 PCs (in UK Biobank) and first 4 PCs (in ELSA) to control for varying levels of population stratification present in the samples.

#### Association between hippocampus volume PRS and MDD traits

As hippocampal volumetric differences have been more closely associated with recurrent MDD and early illness onset,^5, 36^ hippocampus PRS regression analyses were also run with recurrent MDD, number of episodes, MDD duration and age of onset as dependent variables (for further details see Supplementary Materials). In GS:SFHS these were run utilising mixed linear model analysis (as above) to account for the family structure. As recurrent MDD is a binary trait, this was transformed from the linear to liability scale using the Taylor series approximation^35^ (as above). For testing association in unrelated samples, logistic regression models were used for binary traits (recurrent MDD) and linear regression for quantitative traits (number of episodes, MDD duration and age of onset). Models were adjusted for age, sex and the first 15 PCs to control for population stratification. These data were not available for ELSA therefore this was run in UK Biobank only.

#### Meta-analysis

In order to increase power, fixed effect meta-analysis, weighted by standard error of the beta values relating PRS scores to MDD was carried out using the ‘meta’ package (version 4.3-2)^37^ in R.

### Mendelian randomisation

Mendelian randomisation (MR) is an approach that examines genetic variants in association with an exposure and outcome to determine causality. In this study if a significant genetic correlation (*P*<0.05) was found (indicating pleiotropy) it was carried forward into a two-sample MR analysis. We utilised the ‘MendelianRandomization’ package (v0.2.0) in R to conduct both an Inverse-Variance Weighted (IVW) analysis and MR-Egger regression.^21^ In brief, the IVW method incorporates multiple SNPs as a vector of instrumental variables (IVs) and carries out weighted linear regression analysis between the IVs vector—outcome and IVs vector—exposure. The analysis is weighted on the inverse variance of the IVs vector—outcome association and the intercept constrained to zero. We utilised the effect beta from the genome-wide significant variants from the original ENIGMA GWAS as the association between variants and exposure and the effect beta from the same variants in the PGC GWAS as the association between the variant and outcome. We also tested the association between the variants and MDD in the GS:SFHS, UK Biobank and ELSA (Supplementary Materials). If the variant was not available in a data set, that data set was either removed or the variant in highest LD available in both data sets was used. The constraint of the intercept at zero in IVW, however, assumes that all IVs are valid. As this is not always the case, if a significant association (*P*<0.05) was indicated in IVW analysis, sensitivity analysis with MR-Egger regression was conducted. As MR-Egger regression does not constrain the intercept, it is therefore is not biased by invalid IVs.^38^ The same effect beta’s and standard errors were utilised in the MR-Egger regression. For further details on the methodology see Supplementary Materials.

### BUHMBOX

To further explore correlation between subcortical volume and MDD, we utilised the technique BUHMBOX.^22^ This technique tests for the presence of true pleiotropy and genetic subgroup heterogeneity within cases of a disease phenotype (phenotype A) by measuring pairwise correlations of risk alleles with another trait (phenotype B). The presence of phenotype B risk alleles across all phenotype A cases and not phenotype A controls provides evidence of true pleiotropy, whereas subgroup heterogeneity is implicated if phenotype B risk alleles are enriched in a subgroup of phenotype A cases. Pairwise correlations are combined to generate a BUHMBOX test statistic for clinical heterogeneity. In this study, if a significant genetic correlation (*P*<0.05) was found, we utilised BUHMBOX to further dissect the genetic relationship between regional brain volume and MDD. We examined risk alleles associated with the ENIGMA regional brain volumes as phenotype B with MDD phenotypes in GS:SFHS, UK Biobank and ELSA as phenotype A. In order to minimise bias caused by related individuals in GS:SFHS, an unrelated subsample was used comprising 5659 individuals (786 MDD cases). LD pruning was conducted using PLINK 1.90^39^ using —indep-pairwaise with *r*^2^>0.1 and a window size of 50 SNPs and a sliding winding of five SNPs. The first 4 PCs were fitted in GS:SFHS and ELSA and the first 15 PCs were fitted in UK Biobank to account for additional heterogeneity.

### Power Analyses

Power analyses for the genetic correlations (*r*_G_), calculated using LD score regression, were completed using the GCTA-GREML power calculator.^40^ As LD score regression utilises summary statistics and GCTA, the individual genotype data, true power is likely to be slightly lower; however, the GCTA-GREML power calculator gives a close estimate. Results of the power analysis are presented in Supplementary Table S1.

**Figure 1:**
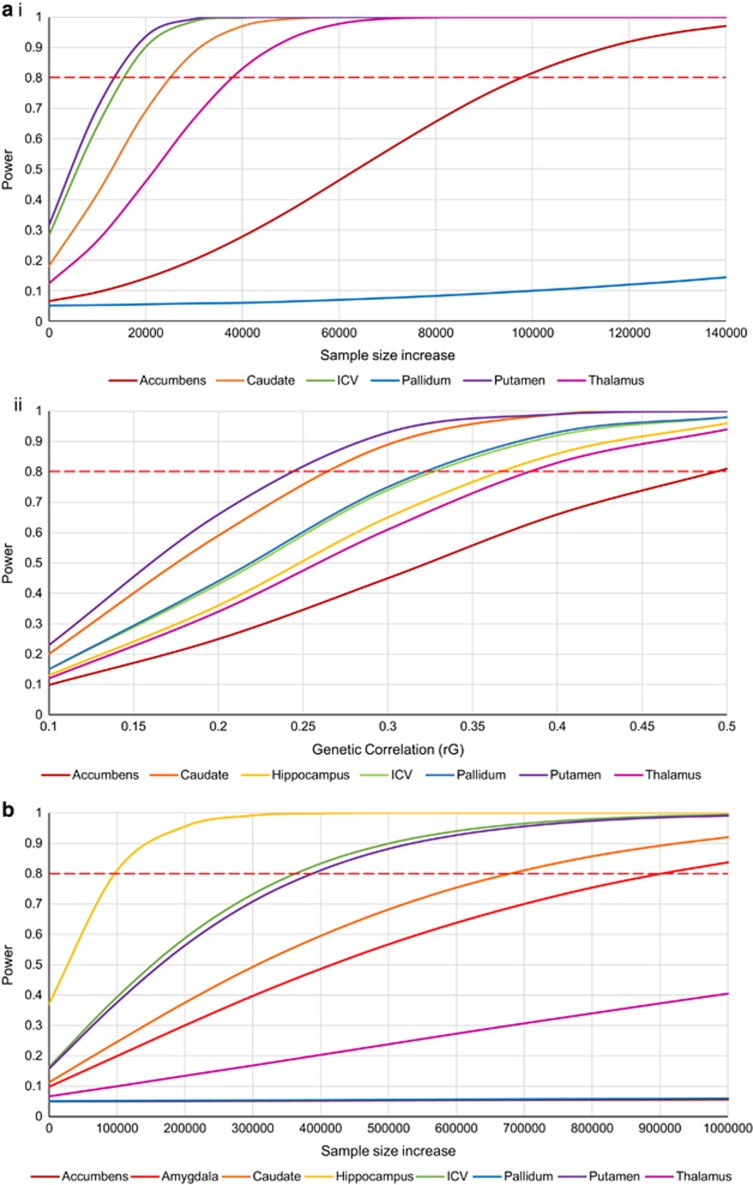
Power curves were calculated with starting point 0 as the sample size in our analysis. For the genetic correlation power analysis the power curves demonstrate (**a**(i)) the sample size increase needed for detecting a significant association at the estimates reported in this analysis when both samples were increased equally and, for MDD, the proportion of cases and controls was kept constant and (**a**(ii)) the genetic correlation that there would be power to detect at the sample size reported in this analysis. For PRS power analysis (**b**) the sample size for the training set (ENIGMA) was kept constant while the target set sample size was increased. Amygdala was assumed to have a *r*_G_=0.25 for the PRS power analysis. Hippocampus had adequate power in the genetic correlation analysis and therefore was not included in the power curve. ICV, intracranial volume; MDD, major depressive disorder; PRS, polygenic risk scoring.

Simulations of genetic correlations varying from 0.1 to 0.5 between the brain volume and MDD indicated that, at *r*_G_=0.5, we had power to detect an association (*P*<0.05) in all regions. However, genetic correlation was found to be much lower for many of the regions and therefore we only had adequate power to detect a correlation between hippocampal volume and MDD (power=93%). For the remaining regions, a power curve was conducted to demonstrate the size of the sample needed for sufficient power (Figure 1 and Supplementary Figure S2). Results indicate that an additional ~15 000 sample increase in both ENIGMA and PGC samples would be needed to detect significant genetic correlations between MDD and either putamen or ICV at the estimates reported in this analysis. To demonstrate significant genetic correlation between MDD and either nucleus accumbens or pallidum volumes would require sample size increases of greater than 100 000 in both samples. Power curves of simulated genetic correlations (varying from 0.1 to 0.5) were also constructed to identify the genetic correlation that we would have had power to detect at the current sample size. Results indicate that this sample had adequate power to detect a genetic correlation of at least 0.24 for putamen, 0.26 for nucleus caudate, 0.33 for both pallidum and ICV, 0.37 for hippocampus, 0.38 for thalamus and 0.49 for nucleus accumbens.

Power analysis of PRS were completed using AVENGEME.^41^ Markers were assumed to be independent and 5% of SNPs were assumed to have an effect in the training sample. Genetic covariance values were taken from the LD score regression analysis, however, no value could be computed for amygdala therefore three hypothetical covariances were tested; 0.50, 0.25 and 0.10. Results of the power analysis are presented in Supplementary Table S3.

Despite a study size of 49 576 individuals, PRS was under-powered in all analyses. Highest power in the meta-analyses was for hippocampal volumes (37%). Low SNP heritability and low covariance between traits account for the low power in the meta-analyses. In the PRS analysis on their own trait, highest powered was the putamen (23%) at a *P-*value threshold of 1. In this analysis a small sample size of 968 individuals likely reduced power. We therefore conducted a power curve for both the meta-analysis and PRS in their own trait to indicate the sample size that would be necessary to have adequate power (Figure 1 and Supplementary Figure S3). Power curves indicate that a sample increase of ~100 000 individuals in the target set would be sufficient power for hippocampus PRS associated with MDD, however nearly 900 000 for amygdala assuming a covariance of 0.25 and an increase of over 1 million participants for nucleus accumbens, pallidum and thalamus.

## Results

### Genetic correlation

Using LD score regression, we calculated SNP-based heritability estimates for the seven subcortical regions and ICV with MDD, utilising summary data from GWAS completed by ENIGMA^17^ and PGC,^33^ respectively. The estimate of the SNP heritability for the amygdala was non-significant and therefore the amygdala was not included in any further analysis. SNP heritability estimates for the remaining subcortical volumes ranged from the SNP *h*^2^=0.0855 (s.e.=0.0438) for the nucleus accumbens to SNP *h*^2^=0.297 (s.e.=0.051) for the putamen (Table 1). MDD SNP heritability was calculated at 0.204 (s.e.=0.0386). Genetic correlation between each subcortical region and ICV with MDD was then calculated. Hippocampal volume demonstrated significant genetic correlation with MDD (*r*_G_=0.460, s.e.=0.200, *P*=0.0213; Table 1), although this did not survive multiple testing correction using false discovery rate adjustment (Supplementary Table S3). No other subcortical volume or ICV was genetically correlated with MDD.

### Polygenic risk score

#### Association between regional brain volume PRS and its respective volume

**Figure 2:**
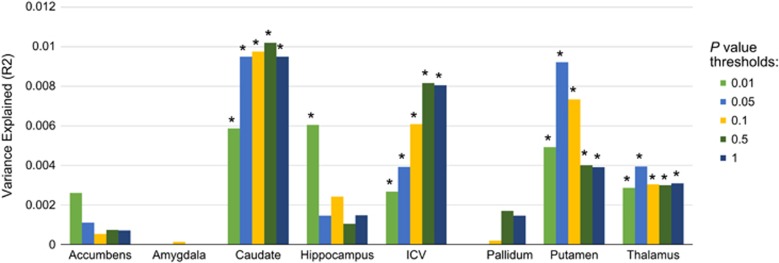
Significant *P-*values (<0.05) are indicated with asterisk (*). Nucleus accumbens, amygdala and pallidum PRS were not significantly associated with their respective volume at any threshold. ICV, intracranial volume; PRS, polygenic risk scoring.

Subcortical and ICV PRS were calculated in UK Biobank to examine the association between each regional volume PRS and its own volume. PRS were positively associated with their respective volume in four of the eight structures across the five *P-*value thresholds; caudate nucleus, ICV, putamen and thalamus. In addition, hippocampus was significantly associated at a *P*-value threshold of 0.01 only. These results retained significance after multiple test correction across the five thresholds, however only raw *P*-values have been reported. Nucleus accumbens, amygdala and pallidum PRS did not demonstrate any association with their respective volume. The variance explained by PRS was small for all volumes, with the largest reported in the caudate nucleus (*R*^2^=0.0102, *β*=0.117, *P*=1.08 × 10^−4^; Figure 2 and Supplementary Table S2).

#### Association between regional brain volume PRS and MDD

**Figure 3:**
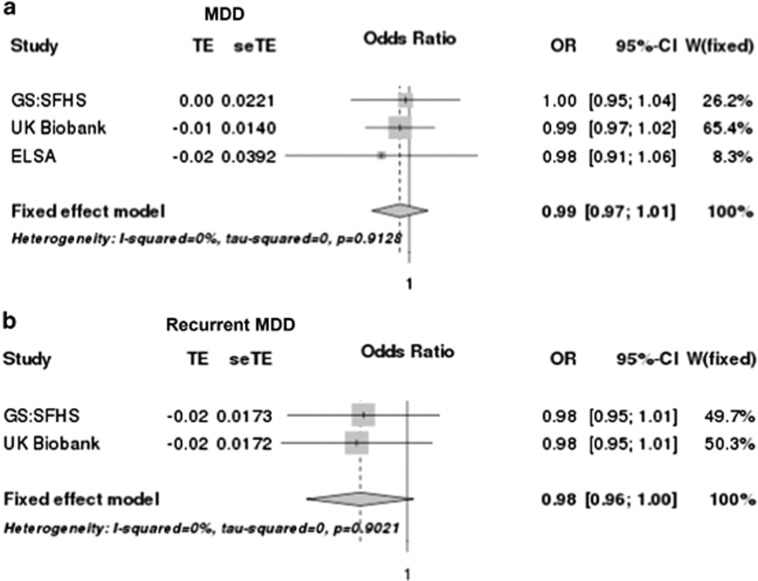
Both plots demonstrate a negative correlation with MDD and recurrent MDD with no heterogeneity between cohorts but neither plot reaches statistical significance. CI, confidence intervals; GS:SFHS, Generation Scotland: Scottish Family Health Study; MDD, major depressive disorder; OR, odds ratio; seTE, standard errors; TE, treatment effect (regression beta’s); W(fixed), weight of individual studies in fixed effect meta-analysis.

Structural PRS were selected at the threshold that best predicted its own volume (nucleus accumbens=0.01, amygdala=0.1, caudate nucleus=0.5, hippocampus=0.01, ICV=0.5, pallidum=0.5, putamen=0.1, thalamus=0.05) and tested for prediction of MDD status. No PRS for any volume was significantly associated with MDD status in any of the cohorts (Supplementary Table S4). In order to increase power, we completed a meta-analysis of the summary association statistics from three cohorts. No evidence of heterogeneity was identified in any of the meta-analyses. We found no association between any structural PRS and MDD (Figure 3a and Supplementary Figure S4).

#### Association between hippocampus volume PRS and MDD traits

Association between hippocampal volume and recurrent MDD and early illness onset has been previously reported.^5, 36^ We therefore examined MDD phenotypes in association with hippocampal volume PRS in GS:SFHS and UK Biobank, these data were not available for the ELSA cohort. There was no association between hippocampal PRS and recurrent MDD (OR=0.98, *P*=0.0850) (Figure 3b). Further, hippocampal volume PRS was not significantly associated with number of episodes (*β*=−0.00390, *P*=0.425), MDD duration (*β*=−0.00110, *P*=0.414) or age of onset (*β*=0.0142, *P*=0.291; Supplementary Figure S5).

### Mendelian randomisation

To further examine the nominally significant genetic correlation between hippocampus and MDD, MR analysis was performed to test for a directional association between hippocampal volume and MDD. Only two genome-wide significant variants were identified in the original ENIGMA GWAS (rs61921502 and rs77956314) and these SNPs were only present in two of the cohorts (GS:SFHS and UK Biobank). To obtain a value for variant—outcome in the PGC data set, we selected the SNPs in highest LD with the two causal variants that were available in both ENGIMA and PGC summary statistics; rs17765551 and rs7294919. We also conducted the analysis using values obtained with the causal variants from meta-analysis with GS:SFHS and UK Biobank. IVW analysis did not identify evidence for a causal association between hippocampus variants and MDD in using either PGC MDD as the outcome nor GS:SFHS and UK Biobank MDD (Supplementary Table S5), it was therefore not necessary to carry this forward into the MR-Egger regression model.

### BUHMBOX

To further investigate whether the genetic correlation found between hippocampus volume and MDD could be due to genetic subgroup heterogeneity, we utilised the software package BUHMBOX. SNP subsets were used that were associated with hippocampal volume at a threshold of *P*<1.0 × 10^−3^ and had to be present in all individuals per cohort therefore 388, 504 and 386 SNPs, and 5659, 7017 and 4118 individuals remained in GS:SFHS, UK Biobank and ELSA, respectively. Clinical heterogeneity was found in GS:SFHS MDD cases (*Z-*score=2.78, *P*=0.00281), demonstrating excessive pairwise correlations between risk alleles for hippocampus volume and a subgroup of MDD cases. This survived false discovery rate multiple testing correction, however, this finding was not replicated in either UK Biobank or ELSA (Table 2).

## Discussion

Previous studies have reported phenotypic associations between brain volumes and MDD. In this study, we investigated whether there was evidence of shared genetic architecture between subcortical brain volumes and ICV with MDD. Results from the genetic correlation analysis indicate that hippocampal volume and MDD are partially influenced by common genetic variants (*r*_G_=0.46, s.e.=0.200, *P*=0.0213), although this did not survive correction for multiple testing. This positive genetic correlation is novel, so far as any of the authors are aware, however Mathias *et al.*^42^ demonstrated significant negative correlation between recurrent MDD and right hippocampal volume measured via linkage analysis in a sample of 893 individuals. No other brain regions’ volume showed evidence of shared genetically aetiology with MDD. Our sample size was adequate to detect a correlation at *r*_G_=0.5, however, at the values reported in this study, we were underpowered in all other brain volumes (excluding hippocampus). Power analyses indicate that we had insufficient power to detect weak to modest genetic correlations in this study. Although we were able to demonstrate lack of strong genetic correlations between regional brain volumes (excepting hippocampus) and MDD, we cannot with confidence exclude the possibility that they are weakly to modestly correlated. Therefore, further analysis utilising larger sample sizes (a minimum of 15 000 increase in both samples) would be necessary to draw confirmatory conclusions.

Brain volume PRS were not associated with their own volume in three out of the eight structures and was only associated with hippocampal volume at *P-*value threshold 0.01. This is likely due to the analysis being underpowered to detect an association in a sample size of 968 participants. Of the PRS that were associated with their phenotype, the largest proportion of variance explained was 1% with the majority predicting ~0.6%. The proportion of variance explained is therefore very low although this is fairly common in PRS studies^43^ with one of the largest explained variance by PRS reported in schizophrenia (~7% on the liability scale).^44^

Meta-analysis of data from three studies, totalling 49 576 individuals including 11 552 cases, found no evidence of association between any regional brain volume PRS and MDD, including the hippocampus. As previous neuroimaging evidence suggests that decreased hippocampal volumes could occur as a cause or consequence of recurrent depressive episodes and early illness onset,^5, 36^ we examined hippocampal volume PRS associations with recurrent MDD, number of episodes, MDD duration and age of onset but we observed no significant associations. Despite the PRS meta-analysis being the largest analysis to date examining genetic scores for brain volume and MDD, it was severely underpowered; therefore, we can draw no confirmatory conclusions about the genetic overlap between any structure and MDD from this analysis. The apparent discrepancy between PRS and our finding LD score regression is likely due to this lack of power, however, as LD information is utilised in LD score regression and SNPs are pruned in PRS calculation, it is also possible that the ‘loss of information’ involved in calculating PRS contributed. Previous simulation studies have demonstrated that predictive capabilities of PRS are greatly enhanced when utilising LD information.^45^ This implies that LD pruning may be removing causal SNPs and those more closely tagging causal variants, resulting in a loss of information and predictive accuracy.

To further dissect the positive genetic correlation between hippocampus and MDD, we utilised MR and BUHMBOX techniques. MR was used to determine the causality of genetic variants in association with hippocampal volume and MDD. We did not detect a causal association, however, there were only two genome-wide significant SNPs associated with hippocampal volume in the original GWAS. Larger numbers of associated variants increase power in MR analysis^38^ therefore this was likely a contributing factor in this analysis. We also applied BUHMBOX to investigate genetic subgroup heterogeneity and detected evidence of a subgroup in MDD cases within GS:SFHS. We did not replicate this finding in UK Biobank or ELSA, however, MDD cases are not defined using a clinical measure in these cohorts, whereas GS:SFHS cases are defined using DSM-IV criteria. The PGC MDD definition also most closely matches that of GS:SFHS MDD, although the GS:SFHS sample was population-based rather than identified from a clinically ascertained samples. This could explain why the findings were associated with these cohorts and not the others. The observed lack of replication may then be due to factors related to ascertainment differences and should therefore be replicated in a clinically determined MDD sample.

We conclude that hippocampal volume and MDD may share common genetic factors, although this result did not withstand multiple testing correction. Animal models have previously demonstrated that increased stress can drive decreased hippocampal neurogenesis (and therefore increased atrophy)^46^ and this reduced neurogenesis can lead to depressive-like symptoms.^47^ Stress is a well-established environmental risk factor associated with MDD^48^ and the inhibition of glucocorticoid receptors has been shown to normalise hippocampal neurogenesis^49^ and relieve symptoms in psychotic major depression.^50^ Furthermore, increased duration of depression has also been related to more pronounced hippocampal reductions.^51^ Our results however indicated a positive genetic correlation suggesting that genetic variants determining larger hippocampal volume may be risk factors for MDD. The clinical heterogeneity found utilising BUHMBOX in GS:SFHS could provide a possible explanation for the deviation from literature. If genes for larger hippocampal volume are present in a subgroup of MDD only, then it is possible that hippocampal volume atrophy could be associated with a different subgroup of individuals that are affected through more environmental pathways. Hippocampal volume has been demonstrated to be more highly impacted by the environment than other brain regions^52^ and is associated with many environmental factors, for example, stress,^48^ increased exercise training^53^ and jet lag.^54^ It is therefore possible that the previously reported decreased hippocampal volume associated with MDD is due to multiple episodes of depression and that this positive genetic correlation is due to a role in MDD susceptibility earlier in brain development. In fact, it has been previously demonstrated that first episode MDD subjects exhibited marginally larger hippocampal volumes in comparison to healthy controls.^55^ This could also provide explanation for the opposing negative genetic correlation finding by Mathias *et al.*^42^ as they examined recurrent MDD. However, it should be noted that this study did not demonstrate significant hippocampal atrophy in analysis including controls,^55^ which has been similarly shown in another study.^56^ Given that this positive correlation could be associated with a subgroup of MDD cases, it is also possible that this is hindering investigations into hippocampus and all MDD cases. Hippocampal volume changes are also widely associated with other psychiatric disorders such as schizophrenia. A similar analysis that examined the genetic correlation between subcortical volumes and schizophrenia found no significant correlations.^57^ This is suggestive that the genetic correlation observed could be specific to hippocampal volume in MDD. However, these results are only indicative of a genetic correlation between the two traits and further research would be necessary to provide confirmative evidence.

A number of limitations of this study should be noted; first, this study only explored the effects of common genetic variants and it may be important to examine rarer variants to generate a more complete picture, although this will require larger sample sizes. Second, the lower heritability, higher prevalence and likely heterogeneity of MDD results in less precise estimates of marker weights from GWAS,^58^ decreasing the power to detect genetic correlations with other phenotypes. Power of the PRS is limited also by the size of the initial ENIGMA GWAS (*n*=11 840), larger discovery sample sizes greatly improve the accuracy of PRS.^44, 59^ Therefore, larger genome-wide analysis would be necessary to generate confirmatory conclusions. Third, the estimates for SNP heritability, calculated using LD score regression, were lower than have been previously described.^60^ LD score regression has been utilised previously to calculate SNP heritability of subcortical volumes using the ENIGMA summary data with similar low estimates reported.^57^

Despite these limitations, we provide some evidence of a positive genetic correlation between hippocampal volume and MDD and an indication of MDD subgroup heterogeneity, however, the genetic correlation did not survive multiple testing correction and the subgroup heterogeneity was not replicated. We did not demonstrate an association utilising PRS techniques, however, low power, low explanation of variance and loss of LD information were notable limitations. Although we demonstrate a potential genetic relationship between hippocampal volume and MDD in a subgroup of individuals, we believe one of the most important outcomes for the current study is in the planning for future studies. Sample sizes of ~150 000 individuals will be needed to have sufficient statistical power (>0.8) to detect shared genetic architecture between MDD and hippocampal volume using PRS, using data sets similar to the one studied. The other regional brain volumes ranged from needing an additional sample size of ~400 000 to in excess of 1 million individuals. Alternatively, further studies may utilise data from further releases of the ENIGMA consortium, including larger numbers of participants and more accurately determined SNP effect sizes. Further research into subgrouping in the association between hippocampus and MDD may also be beneficial.

## Figures and Tables

**Table 1:** SNP-based heritability (h2) and genetic correlation (*r*_G_) of subcortical brain regions and ICV with MDD

	*SNP heritability*			*Genetic correlation with MDD*			
*Brain region*	*SNP* h^*2*^	*s.e.*	Z*-score*	r_*G*_	*s.e.*	Z*-score*	P
Nucleus accumbens	0.0855	0.0438	1.95	0.0458	0.210	0.218	0.828
Caudate nucleus	0.253	0.0432	5.86	0.0752	0.130	0.580	0.562
Hippocampus	0.137	0.0481	2.85	0.460	0.200	2.30	0.0213
ICV	0.167	0.0462	3.61	0.123	0.166	0.739	0.460
Pallidum	0.171	0.049	3.49	−0.0077	0.158	−0.0491	0.961
Putamen	0.297	0.051	5.82	0.0986	0.118	0.834	0.404
Thalamus	0.125	0.0401	3.12	−0.0808	0.177	−0.457	0.648

Abbreviations: ICV, intracranial volume; MDD, major depressive disorder; SNP, single-nucleotide polymorphism.

The heritability of amygdala was nonsignificant and therefore removed from subsequent analysis. The *P*-values shown are uncorrected for multiple testing (for the results corrected for multiple testing see Supplementary Table S2).

**Table 2:** BUHMBOX results for hippocampus volume and MDD in GS:SFHS, ELSA and UK Biobank

	*BUHMBOX P*	P_*corrected*_	Z*-score*	N	*Cases*	*Controls*	*SNPs*N	*Pleiotropy*P	*Pleiotropy*β	*Pleiotropy s.e.*
GS:SFHS	**0.00281**	**0.00843**	**2.77**	**5659**	**786**	**4873**	**388**	0.0890	−0.0448	0.0263
UK Biobank	0.893	0.893	−1.24	7017	2316	4701	504	0.926	−0.00128	0.0138
ELSA	0.678	0.893	−0.462	4116	544	3572	386	0.650	0.0144	0.0316

Abbreviations: GS:SFHS, Generation Scotland: Scottish Family Health Study; ELSA, English Longitudinal Study of Ageing.

Significant results (*P*<0.05) are shown in bold.
