# Supplementary material for: Do regional brain volumes and major depressive disorder share genetic architecture? A study of Generation Scotland (n=19 762), UK Biobank (n=24 048) and the English Longitudinal Study of Ageing (n=5766)
Source: Transl Psychiatry. 2017 Aug 15;7(8):e1205–. doi: 10.1038/tp.2017.148 (PMC5611720; doi:10.1038/tp.2017.148)
Supplement: Supplementary Materials [file tp2017148x1.docx]

**This document contains the supplementary materials for the manuscript by Wigmore et al “Shared Genetic Architecture of Regional Brain Volumes and Major Depressive Disorder in Generation Scotland (n=19,762), UK Biobank (n=24,048) and the English Longitudinal Study of Ageing (n=5766)”.**

**Supplemental Methods**

**Cohort descriptions and MDD phenotype**

**GS:SFHS:** GS:SFHS is a cohort of 24,080 participants collected from Scottish medical practices between 2006 and 2011. Participants were recruited at random and were eligible if they were over 18 years of age and had one first-degree relative also willing to participate. Genotype data was available for 20,032 participants which after QC was reduced to 19,994 (males=8,158, females=11,698). 2,643 participants (13.4%) were given a diagnosis of MDD after SCID interview with 17,119 controls. Episode data was available for 2,643 cases and was coded from 0 to 40 episodes with anyone above that value (including those who had more episodes than they could recall) given the value 41. Age of onset information was available for 2,631 cases. For recurrent MDD, single episode MDD were excluded leaving 1,319 cases. MDD duration was calculated by age of onset subtracted from age of participant.

**UK Biobank:** UK Biobank is an open access cohort of 502,664 individuals collected from across the United Kingdom between 2006 and 2010. Participants were eligible if they were between the age of 40 and 69. After QC, genotype data was available for 152,734. Related individuals and GS and ELSA participants were excluded from this sample leaving 116,909 individuals (males=55,410, females=61,499). 8,146 MDD cases (33.9%) were available in this sample and 15,886 controls, a large number of controls were excluded due incomplete or missing data explaining the higher proportion of cases. MDD episode data was available for 6,466 individuals and coded as for GS:SFHS, therefore, recurrent MDD (excluding any single episode MDD) included 4,612 cases. Age of diagnosis of MDD was available for 1,316 cases and MDD duration derived as described above.

**ELSA:** ELSA is comprised of 12,003 individuals at wave 1 that were collected from the English population. Participants were eligible if they were aged 50 or over. Genotype data was available for 7,412 unrelated individuals which was reduced to 7,230 after QC. 757 MDD cases (13.1%) and 5,009 controls had available genotype data in wave 1 of the data. Wave 1 was selected for analysis as this had the largest sample size of MDD cases (males=2,662, females=3,196). Episode data and age of onset of MDD was not available in ELSA therefore we could not infer recurrent MDD status or MDD duration.

**LD score regression**

Only SNPs that overlapped with HapMap Project Phrase 3 SNPs were included for calculation of SNP heritability and genetic correlations and the following thresholds were applied; INFO > 0.9, MAF > 1%, missingness of 0 and strand-ambiguous SNPs were excluded. For further details on this method please see original paper^1, 2^. The intercept in this analysis was not constrained and multiple comparison testing was completed utilising false discovery rate (FDR).

**Polygenic Profiling**

To generate PRS in each cohort, SNPs were excluded on the following thresholds; MAF < 1%, Hardy Weinberg equilibrium p>1x10^-6^, missingness per individual <1%, missingness per SNP <1%. Principle components were generated utilising the genome-wide complex trait analysis (GCTA) tool ^3^ to control for population stratification and strand-ambiguous SNPs were removed. Clump based LD pruning was performed (r^2^ 0.25, 300kb window). For further details on this method please see original paper^4^.

**Taylor Series Transformation**

The Taylor series transformation^5^ is a method of converting a linear beta to an odds ratio (OR) on the liability scale. It uses the formula $OR= \frac{P+beta/(1-P-beta)}{P/(1-P)}$, where P represents the prevalence of the trait (in this case MDD) in the general population and beta is the beta coefficient from the linear association model.

**Mendelian Randomisation (MR)**

*Genetic variants*

Instrumental variables (IVs) were constructed of SNPs that reached genome-wide significant in the ENIGMA GWAS. As hippocampal volume was the only brain structure to demonstrate nominally significant genetic correlation, MR analysis was only completed on this subcortical structure. Two SNPs reached genome-wide significance in the original GWAS; rs61921502 and rs7294919. These SNPs were not available in the MDD PGC GWAS summary statistics and therefore the SNPs in highest LD available in both datasets (ENIGMA and PGC) were selected. rs17765551 was in LD with rs61921502 (R^2^=0.51) and rs77956314 was in LD with rs7294919 (R^2^=0.86).

ENIGMA SNPs (rs61921502 and rs7294919) were present in both GS:SFHS and UK Biobank however not in ELSA. This dataset was therefore excluded. The genetic variants were extracted using PLINK^6^ and coded as 0,1 or 2. These variants were then carried forward into an association analysis.

*Association in GS:SFHS and UK Biobank*

Following the same model used to measure the association between PRS and MDD, variant – exposure association was measured using mixed linear models in AS-Reml-R in GS:SFHS and logistic regression in UK Biobank. Fixed-effect meta-analysis between the 2 datasets for each SNP association was conducted using the ‘meta’ package in R.

*Mendelian Randomisation (MR) analysis*

We conducted MR analysis using the method outline by Bowden et al., (2015) which utilises 2 techniques; the Inverse Variance Weighted (IVW) method and MR-Egger regression^7^. MR makes three assumptions of instrumental variables (IVs); (1) that the IVs are associated with the exposure, (2) that the IVs are not associated with any confounders and (3) that the association between outcome and exposure is conditional on the IVs (e.g. the IVs do not have a direct effect on the outcome). Any deviation from these assumptions would mean the IVs are invalid. Whilst the IVW method considers that the assumptions are true, MR-Egger regression relaxes these assumptions to account for directional pleiotropy. It uses a weaker assumption known as the Instrument Strength Independent of Direct effect (InSIDE) which is the condition that the correlation between IVs – exposure and direct effect of IVs – outcome is zero. If the intercept differs from zero, then the model contains invalid IVs and the IVW estimate will be biased.

**References**

1. Bulik-Sullivan B, Loh P, Finucane H, Ripke S, Yang J, Schizophrenia Working Group of the Psychiatric Genomics Consortium*, et al*. LD Score regression distinguishes confounding from polygenicity in genome-wide association studies. *Nat Genet* 2015a; **47**(3)**:** 291-295.

2. Bulik-Sullivan B, Finucane H, Anttila V, Gusev A, Day F, Loh P*, et al*. An atlas of genetic correlations across human diseases and traits. *Nat Genet* 2015b; **47**(11)**:** 1236-1241.

3. Yang J, Lee S, Goddard M, Visscher P. GCTA: a tool for genome-wide complex trait analysis. *Am J Hum Genet* 2011; **88**(1)**:** 76-82.

4. International Schizophrenia Consortium, Purcell SM, Wray NR, Stone JL, Visscher PM, O'Donovan MC*, et al*. Common polygenic variation contributes to risk of schizophrenia and bipolar disorder. *Nature* 2009; **460**(7256)**:** 748-752.

5. Cortes A, Hadler J, Pointon JP, Robinson PC, Karaderi T, Leo P*, et al*. Identification of multiple risk variants for ankylosing spondylitis through high-density genotyping of immune-related loci. *Nat Genet* 2013; **45**(7)**:** 730-738.

6. Purcell S, Neale B, Todd-Brown K, Thomas L, Ferreira M, Bender D*, et al*. PLINK: a tool set for whole-genome association and population-based linkage analyses. *Am J Hum Genet* 2007; **81**(3)**:** 559-575.

7. Bowden J, Davey Smith G, Burgess S. Mendelian randomization with invalid instruments: effect estimation and bias detection through Egger regression. *Int J Epidemiol* 2015; **44**(2)**:** 512-525.

**Supplemental Table S1. Power analyses.**

|  | GCTA-GREML Power Calculator | |
| --- | --- | --- |
|  | Simulation rG= 0.5 | Measured rG |
| *Accumbens* | 0.81 | 0.060 |
| *Amygdala* | - | - |
| *Caudate* | 1.00 | 0.13 |
| *Hippocampus* | 0.96 | 0.93 |
| *ICV* | 0.98 | 0.20 |
| *Pallidum* | 0.98 | 0.051 |
| *Putamen* | 1.00 | 0.22 |
| *Thalamus* | 0.94 | 0.097 |

**a)**

**b i)**

|  | PRS in association of own structure | | MDD PRS Meta-Analysis | |
| --- | --- | --- | --- | --- |
|  | PRS thresholds | Power | Covariance | Power |
| *Accumbens* | 0.01  0.05  0.1  0.5  1 | 0.0560  0.0659  0.0730  0.0921  0.0946 | 5% | 0.0503 |
| *Amygdala* | 0.01  0.05  0.1  0.5  1 | 0.0537  0.0602  0.0649  0.0779  0.0797 | 50%  25%  10% | 0.248  0.0980  0.0575 |
| *Caudate* | 0.01  0.05  0.1  0.5  1 | 0.159  0.262  0.318  0.432  0.444 | 8% | 0.113 |
| *Hippocampus* | 0.01  0.05  0.1  0.5  1 | 0.0696  0.0970  0.115  0.161  0.167 | 46% | 0.371 |
| *ICV* | 0.01  0.05  0.1  0.5  1 | 0.0827  0.124  0.151  0.215  0.223 | 12% | 0.163 |
| *Pallidum* | 0.01  0.05  0.1  0.5  1 | 0.0857  0.130  0.159  0.226  0.234 | 1% | 0.0504 |
| *Putamen* | 0.01  0.05  0.1  0.5  1 | 0.227  0.365  0.434  0.558  0.569 | 10% | 0.157 |
| *Thalamus* | 0.01  0.05  0.1  0.5  1 | 0.0654  0.0879  0.103  0.142  0.147 | 8% | 0.0662 |

|  | Meta-analysis | |
| --- | --- | --- |
|  | Covariance | Power |
| *Recurrent* | 50%  25%  10% | 0.264  0.102  0.0581 |
| *Episodes* | 50%  25%  10% | 0.532  0.175  0.0693 |
| *Duration* | 50%  25%  10% | 0.0957  0.0612  0.0518 |
| *Age of Onset* | 50%  25%  10% | 0.0957  0.0612  0.0518 |

**ii)**

GCTA-GREML power calculator was used to calculate (a) power for genetic correlation at the measured correlation and at a simulated rg=0.5. AVENGEME was used to predict power of PRS in (b i) the meta-analysis between subcortical structures and MDD and in prediction of own structure in UK Biobank (b ii) the meta-analysis between depression traits and hippocampal volume. Covariance was measured, were possible, utilising LD score regression between ENIGMA subcortical structures and PGC MDD. If covariance could not be calculated, covariances of 50%, 25% and 10% have been reported. SNP heritability values were calculated from LD score regression were possible, otherwise values from published sources were used. GCTA, genome-wide complex trait analysis; GREML, genomic-relatedness-matrix restricted maximum-likelihood; rG, genetic correlation.

**Supplemental Table S2. Linear regression analysis of polygenic score for regional brain volume and their respective volume in UK Biobank.**

|  |  | **Respective total brain region volume** | |
| --- | --- | --- | --- |
|  | **PRS Threshold** | ***P* value** | **Stats** |
| *Nucleus accumbens* | *0.01*  *0.05*  *0.1*  *0.5*  *1* | 0.0695  0.239  0.412  0.334  0.343 | β= 0.0516  R^2^= 0.00260  β= 0.0333  R^2^= 0.00110  β= 0.0237  R^2^= 0.000531  β= 0.0274  R^2^= 0.000735  β= 0.0270  R^2^= 0.000708 |
| *Amygdala* | *0.01*  *0.05*  *0.1*  *0.5*  *1* | 0.902  0.992  0.703  0.948  0.828 | β= -0.00378  R^2^= 1.36x10^-5^  β= -0.000303  R^2^= 8.5x10^-8^  β= -0.0121  R^2^= 0.000131  β= 0.00212  R^2^= 3.79x10^-6^  β= 0.00707  R^2^= 4.24x10^-5^ |
| *Caudate nucleus* | *0.01*  *0.05*  *0.1*  *0.5*  *1* | **0.00336**  **0.000185**  **0.000151**  **0.000108**  **0.000184** | **β= 0.0795**  **R^2^= 0.00586**  **β= 0.107**  **R^2^= 0.00950**  **β= 0.113**  **R^2^= 0.00975**  **β= 0.117**  **R^2^= 0.0102**  **β= 0.115**  **R^2^= 0.00950** |
| *Hippocampus* | *0.01*  *0.05*  *0.1*  *0.5*  *1* | **0.00417**  0.161  0.0704  0.236  0.157 | **β= 0.0806**  **R^2^= 0.00605**  β= 0.0420  R^2^= 0.00145  β= 0.0536  R^2^= 0.00242  β= 0.0341  R^2^= 0.00104  β= 0.0407  R^2^= 0.00148 |
| *ICV* | *0.01*  *0.05*  *0.1*  *0.5*  *1* | **0.0418**  **0.0134**  **0.00204**  **0.000351**  **0.000388** | **β= 0.0509**  **R^2^= 0.00267**  **β= 0.0649**  **R^2^= 0.00392**  **β= 0.0822**  **R^2^= 0.00609**  **β= 0.0954**  **R^2^= 0.00817**  **β= 0.0947**  **R^2^= 0.00805** |
| *Pallidum* | *0.01*  *0.05*  *0.1*  *0.5*  *1* | 0.902  0.965  0.617  0.131  0.163 | β= -0.00353  R^2^= 1.14x10^-5^  β= -0.00131  R^2^= 1.44x10^-6^  β= 0.0150  R^2^= 0.000186  β= 0.0474  R^2^= 0.00170  β= 0.0437  R^2^= 0.00145 |
| *Putamen* | *0.01*  *0.05*  *0.1*  *0.5*  *1* | **0.00163**  **1.56x10^-5^**  **0.000118**  **0.00451**  **0.00498** | **β= 0.0758**  **R^2^= 0.00492**  **β= 0.118**  **R^2^= 0.00921**  **β= 0.108**  **R^2^= 0.00733**  **β= 0.0878**  **R^2^= 0.00400**  **β= 0.0873**  **R^2^= 0.00391** |
| *Thalamus* | *0.01*  *0.05*  *0.1*  *0.5*  *1* | **0.00602**  **0.00124**  **0.00464**  **0.00513**  **0.00430** | **β= 0.0519**  **R^2^= 0.00286**  **β= 0.0630**  **R^2^= 0.00395**  **β= 0.0556**  **R^2^= 0.00304**  **β= 0.0549**  **R^2^= 0.00300**  **β= 0.0561**  **R^2^= 0.00309** |

***** Five P value thresholds were used; 0.01, 0.05, 0.1, 0.5 and 1. Significant values (*P*<0.05) are shown in bold.

**Supplemental Table S3. Unadjusted and FDR corrected *P* values for LD score regression genetic correlation with MDD.**

|  | **Nucleus accumbens** | **Amygdala** | **Caudate nucleus** | **Hippocampus** | **ICV** | **Pallidum** | **Putamen** | **Thalamus** |
| --- | --- | --- | --- | --- | --- | --- | --- | --- |
| *Unadjusted P value* | 0.828 | NA | 0.562 | **0.0213** | 0.460 | 0.961 | 0.404 | 0.648 |
| *FDR corrected P value* | 0.961 | NA | 0.907 | 0.149 | 0.907 | 0.961 | 0.907 | 0.907 |

The genetic correlation between hippocampus volume and MDD does not withstand FDR correction for multiple testing. Significant values (*P*<0.05) are shown in bold. FDR, false discovery rate.

**Supplemental Table S4. Mixed model analysis of Subcortical volumetric and ICV PRS with MDD in all 3 cohorts.**

| PRS | Study | P value | beta | s.e. |
| --- | --- | --- | --- | --- |
| Nucleus accumbens | **GS** | 0.485 | -0.0181 | 0.0218 |
|  | **UKB** | 0.284 | -0.0151 | 0.0141 |
|  | **ELSA** | 0.659 | 0.0175 | 0.0395 |
| Amygdala | **GS** | 0.327 | -0.0285 | 0.0217 |
|  | **UKB** | 0.962 | 0.000694 | 0.0146 |
|  | **ELSA** | 0.208 | -0.0500 | 0.0398 |
| Caudate nucleus | **GS** | 0.246 | 0.0197 | 0.0224 |
|  | **UKB** | 0.396 | -0.0135 | 0.0159 |
|  | **ELSA** | 0.114 | -0.0630 | 0.0398 |
| Hippocampus | **GS** | 0.782 | -0.00299 | 0.0211 |
|  | **UKB** | 0.601 | -0.00732 | 0.0140 |
|  | **ELSA** | 0.571 | -0.0222 | 0.0392 |
| ICV | **GS** | 0.395 | 0.0179 | 0.0225 |
|  | **UKB** | 0.280 | -0.0160 | 0.0148 |
|  | **ELSA** | 0.0995 | 0.0652 | 0.0396 |
| Pallidum | **GS** | 0.752 | -0.00808 | 0.0221 |
|  | **UKB** | 0.250 | 0.0178 | 0.0155 |
|  | **ELSA** | 0.658 | 0.0176 | 0.0398 |
| Putamen | **GS** | 0.303 | -0.0246 | 0.0218 |
|  | **UKB** | 0.215 | 0.0231 | 0.0186 |
|  | **ELSA** | 0.695 | -0.0161 | 0.0410 |
| Thalamus | **GS** | 0.451 | -0.0192 | 0.0219 |
|  | **UKB** | 0.836 | 0.00293 | 0.0142 |
|  | **ELSA** | 0.222 | -0.0482 | 0.0395 |

* Best *P* value threshold for PRS were carried forward in this analysis; Nucleus accumbens=0.01, Amygdala= 0.1, Caudate nucleus=0.5, Hippocampus=0.01, ICV=0.5, Pallidum=0.5, Putamen=0.1, Thalamus=0.05. No PRS demonstrated a significant association with MDD in any cohort.

**Supplemental Table S5. MR analysis using IVW method.**

|  | **Inverse-Variance Weighted method** | | |
| --- | --- | --- | --- |
|  | ***P* value** | **Beta** | **SE** |
| *Hippocampus-GS&UKB MDD* | 0.361 | 0.0100 | 0.005 |
| *Hippocampus-PGC MDD* | 0.077 | 0.004 | 0.007 |

**Figure S1. Flow chart of methodology.**

**
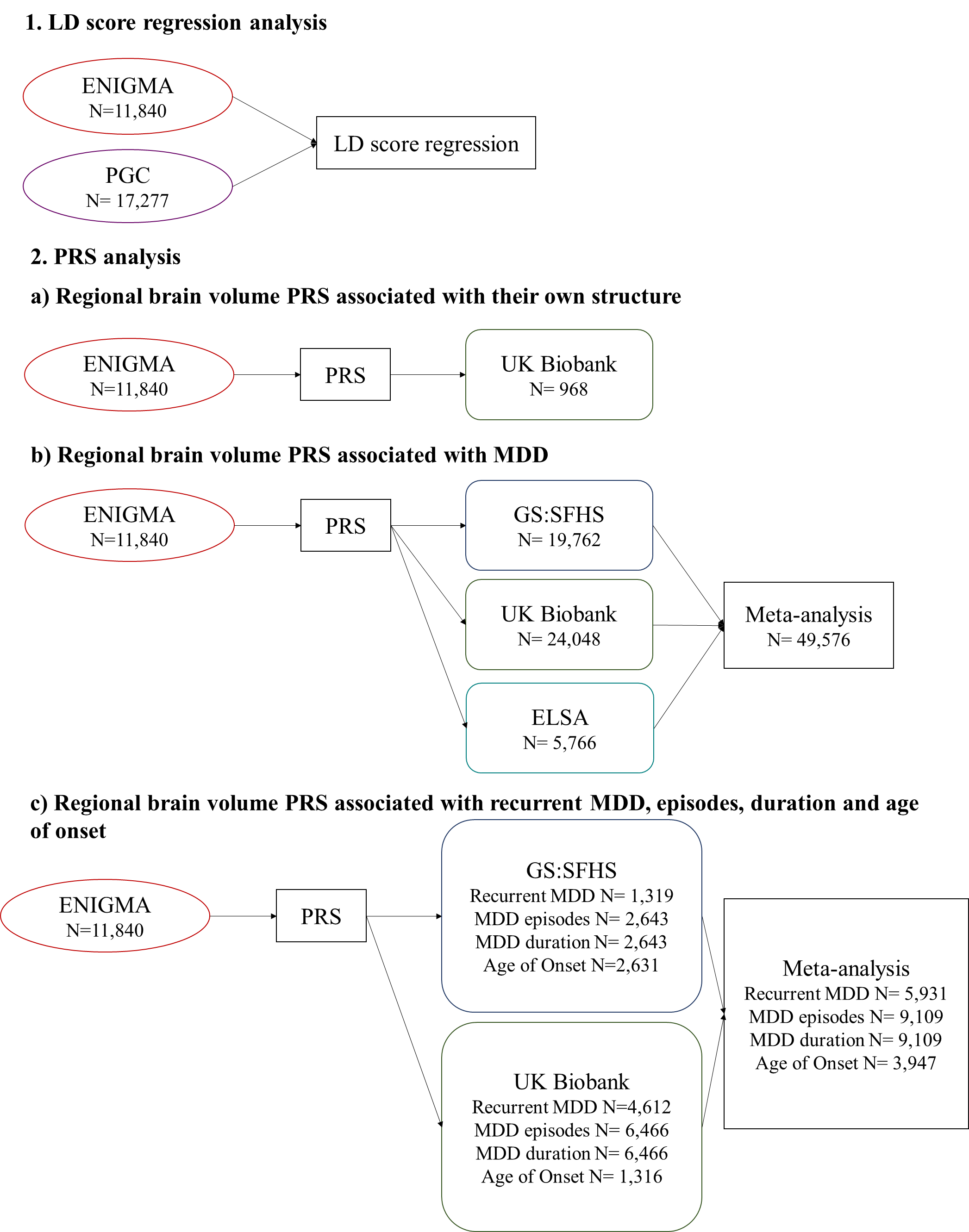
**

**
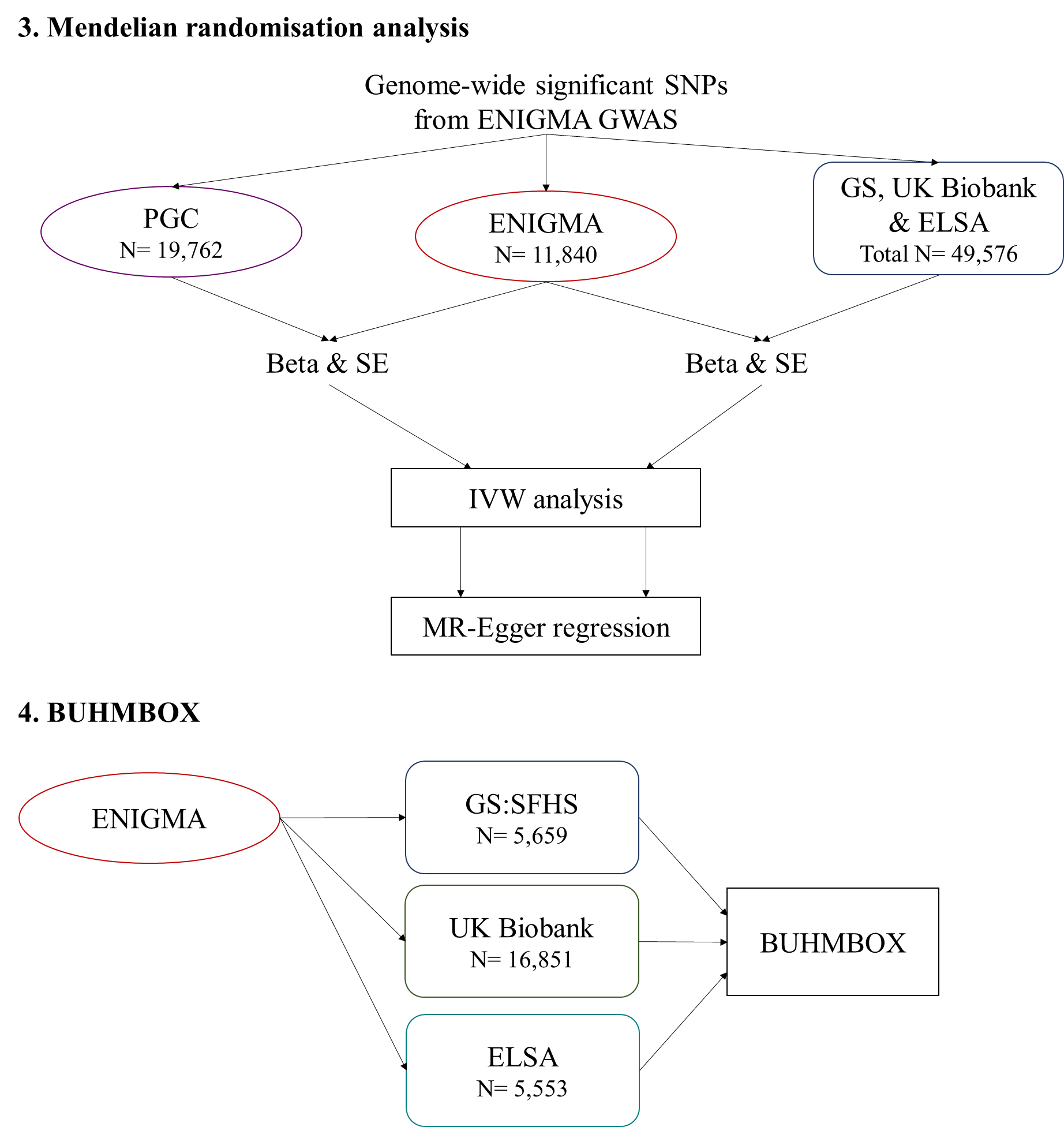
**

**
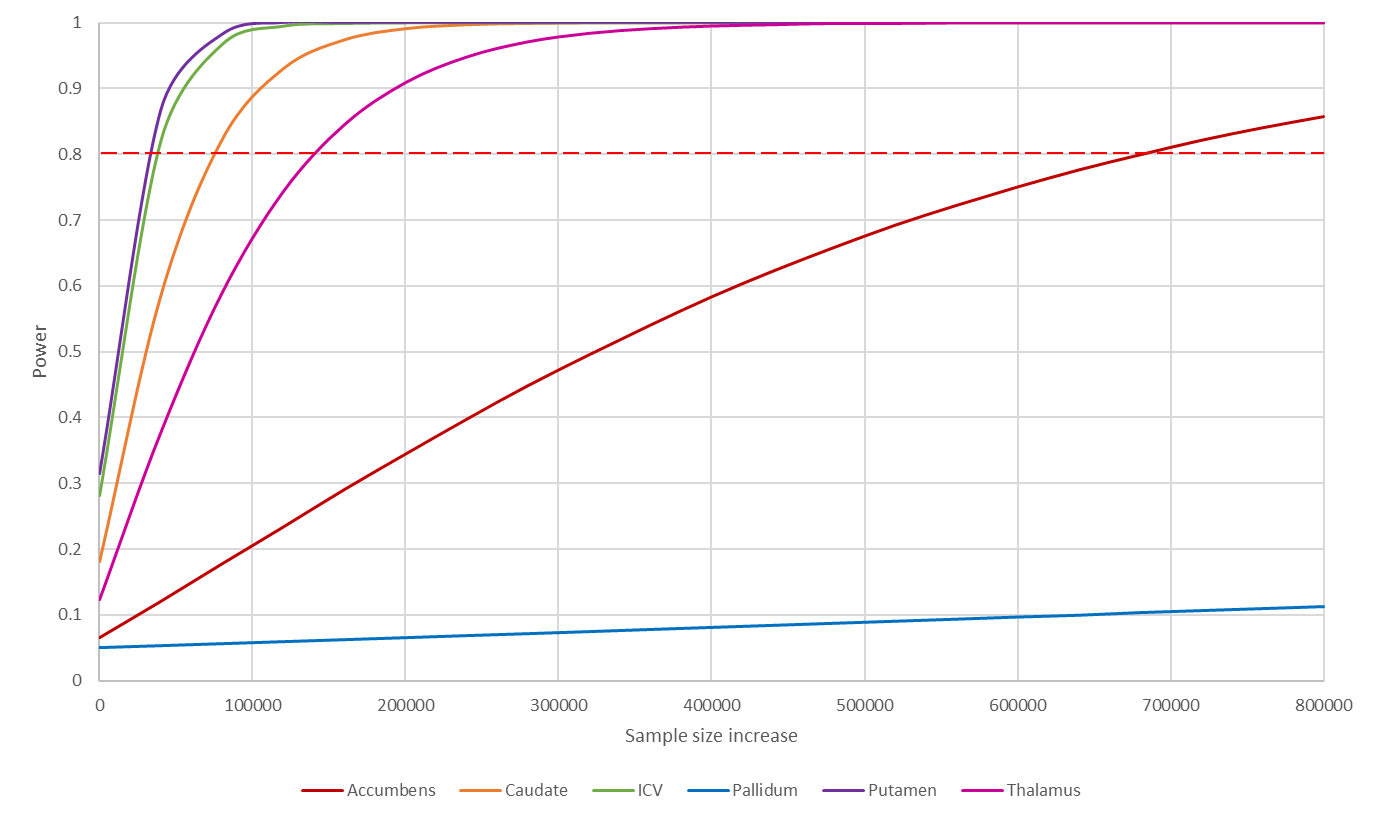
Figure S2. Power curves for genetic correlation analysis a) increasing sample size for subcortical structures only and b) increasing sample size for MDD only**

**
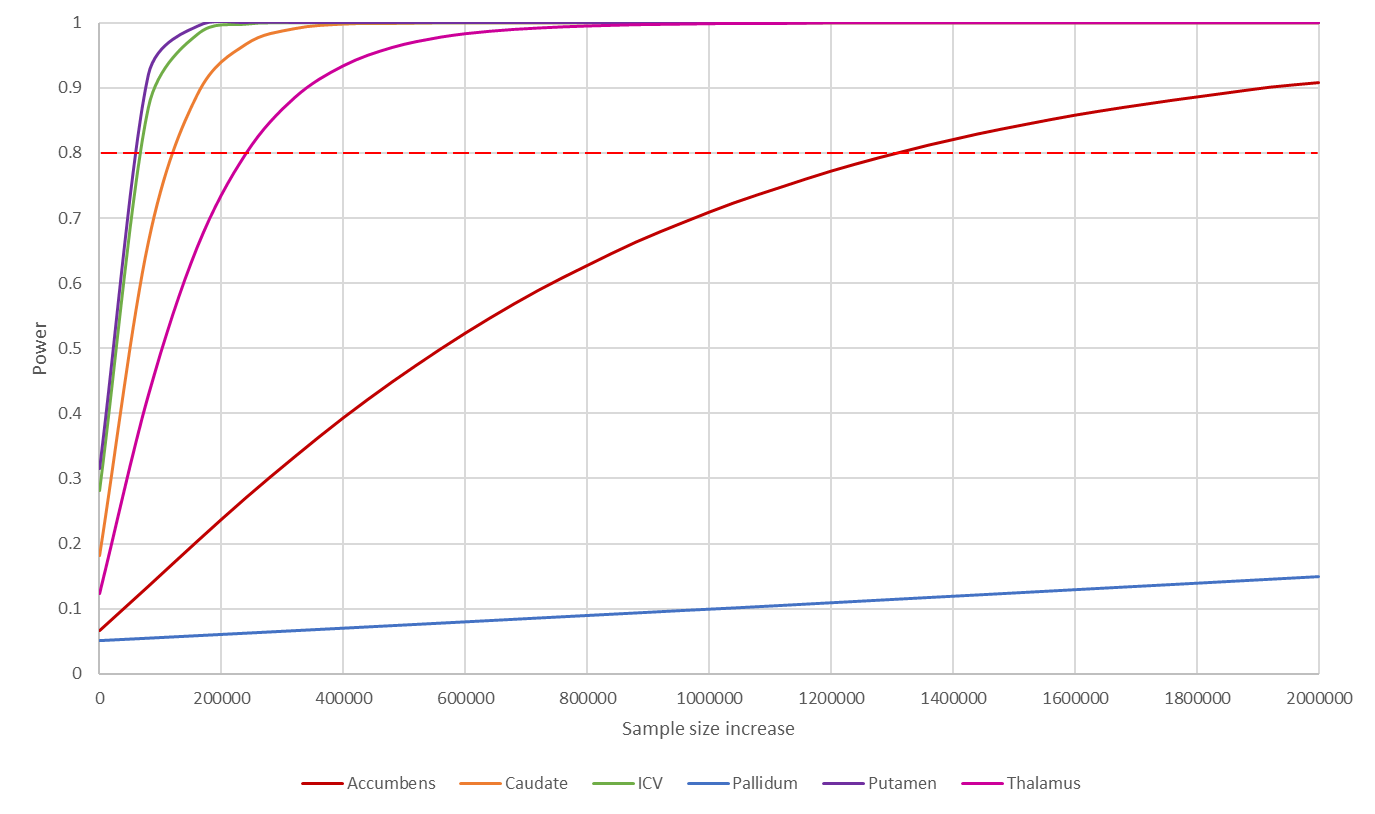
**

Power curves were calculated with starting point 0 as the sample size in our analysis. For increasing the sample for MDD it was assumed the ratio of cases and controls remained the same therefore a sample increase of 1000 would include 500 cases and 500 controls. Hippocampus was not included as this had adequate power.

**Figure S3. Power curves for PRS analyses a) subcortical PRS associated with their respective structures at a P value threshold of (i) 1 and (ii) 0.01 and b) subcortical PRS associated with recurrent MDD, age of onset, episodes and duration.**

1. **
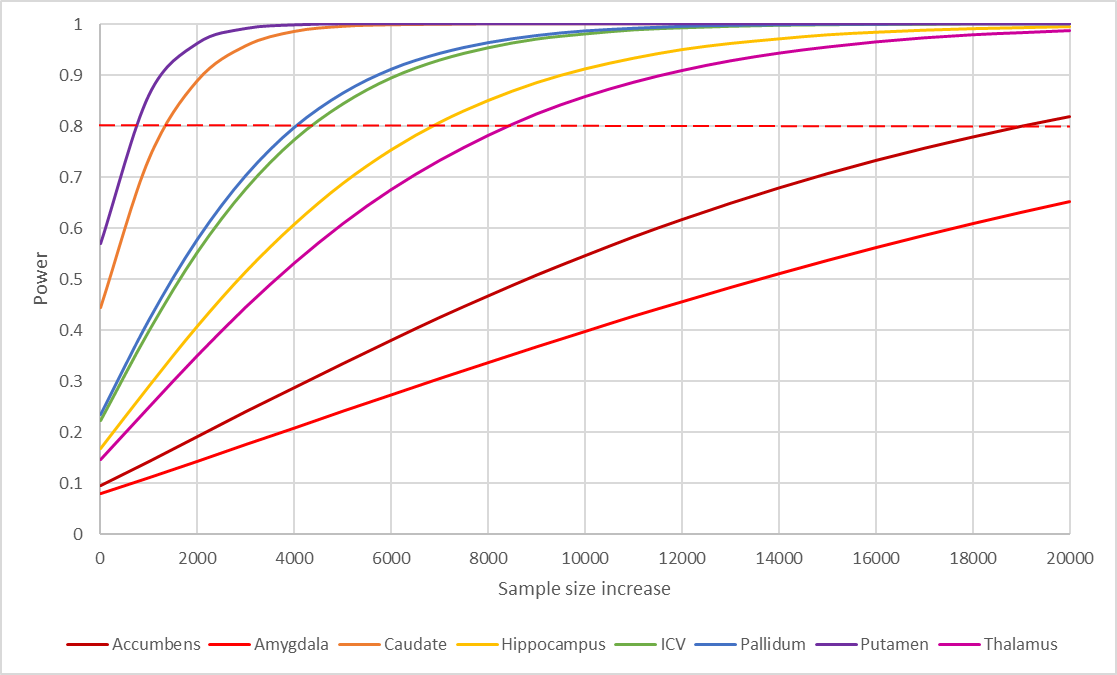
i)**

**
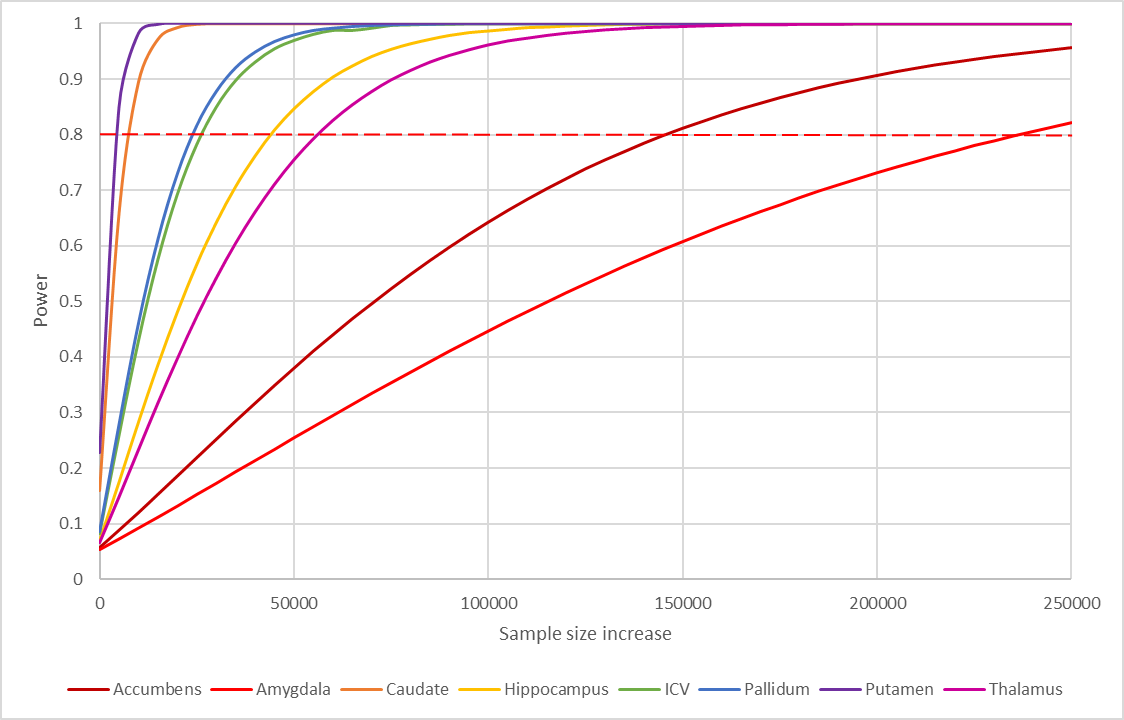
**

**ii)**

1. **
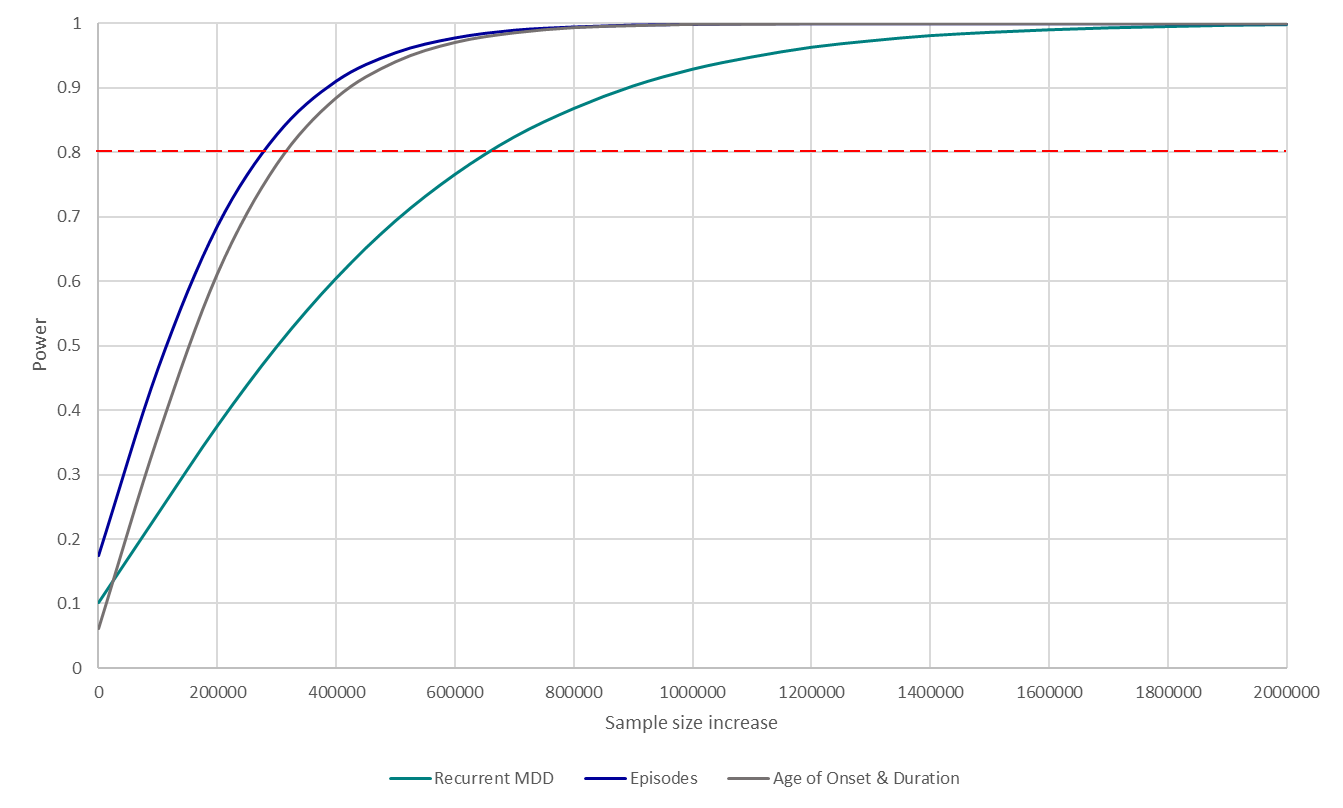
**

Power curves assumed training sample size (ENIGMA subcortical volumes) remained constant and sample size for the target data set was increased from point 0 (the sample at which this analysis was conducted). Amygdala, recurrent MDD, episodes, age of onset and duration were all assumed to have a genetic correlation of 25%.

**Figure S4. Meta-analysis forest plots of mixed model analysis of subcortical volume PRS and MDD in Generation Scotland: Scottish Family Health Study (GS:SFHS), UK Biobank and English Longitudinal Study of Ageing (ELSA).**

**S3. a) Nucleus Accumbens PRS**

**
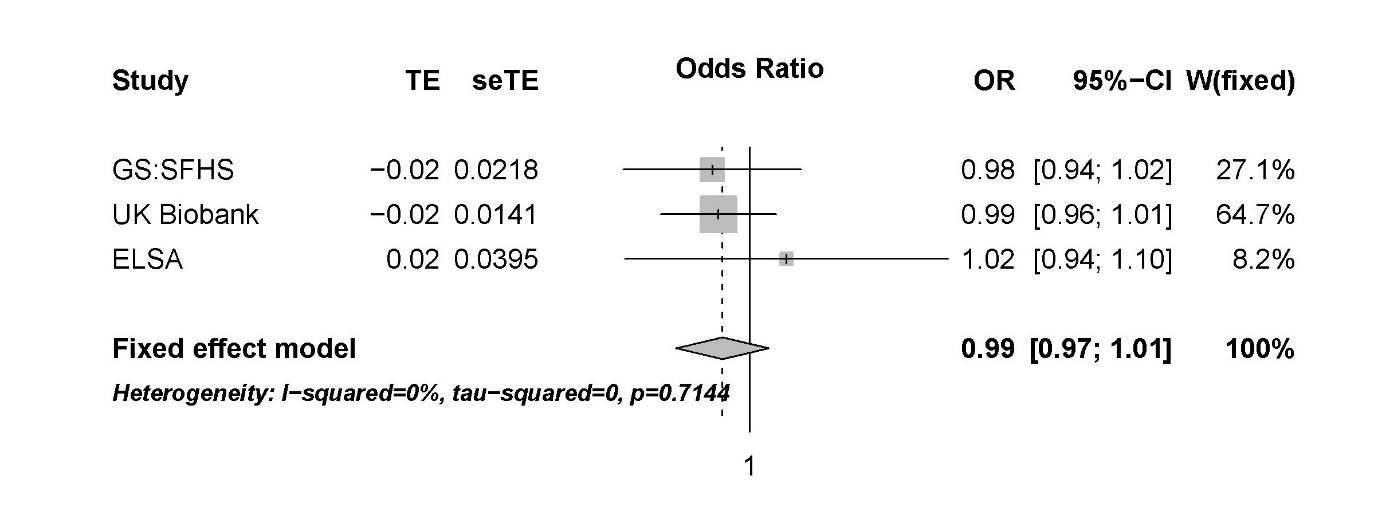
**

**b) Amygdala PRS**

**
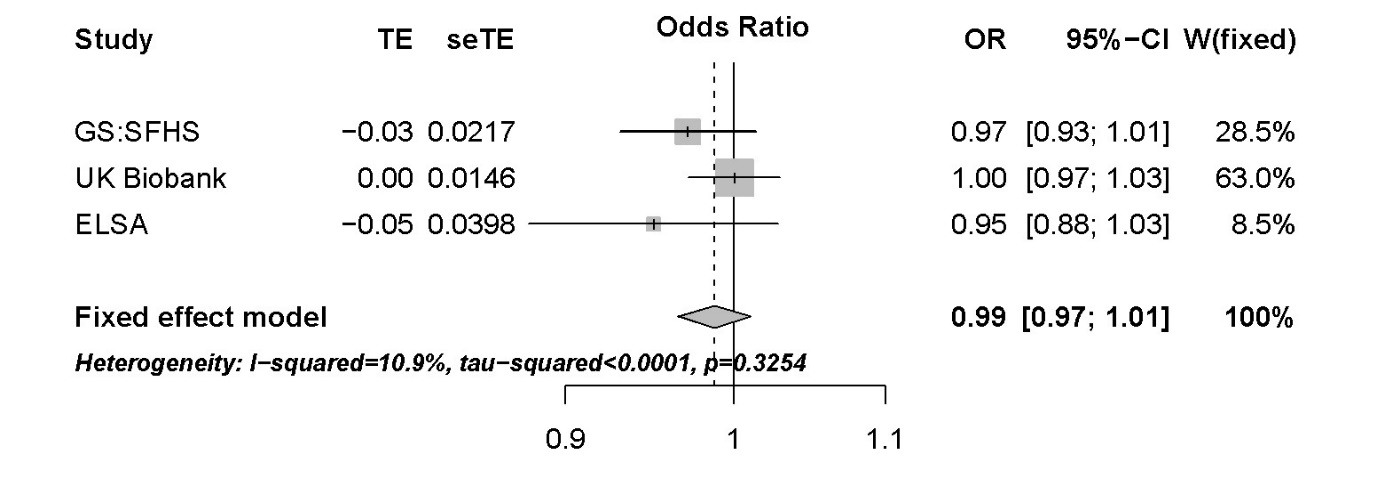
**

**c) Nucleus caudate PRS**

**
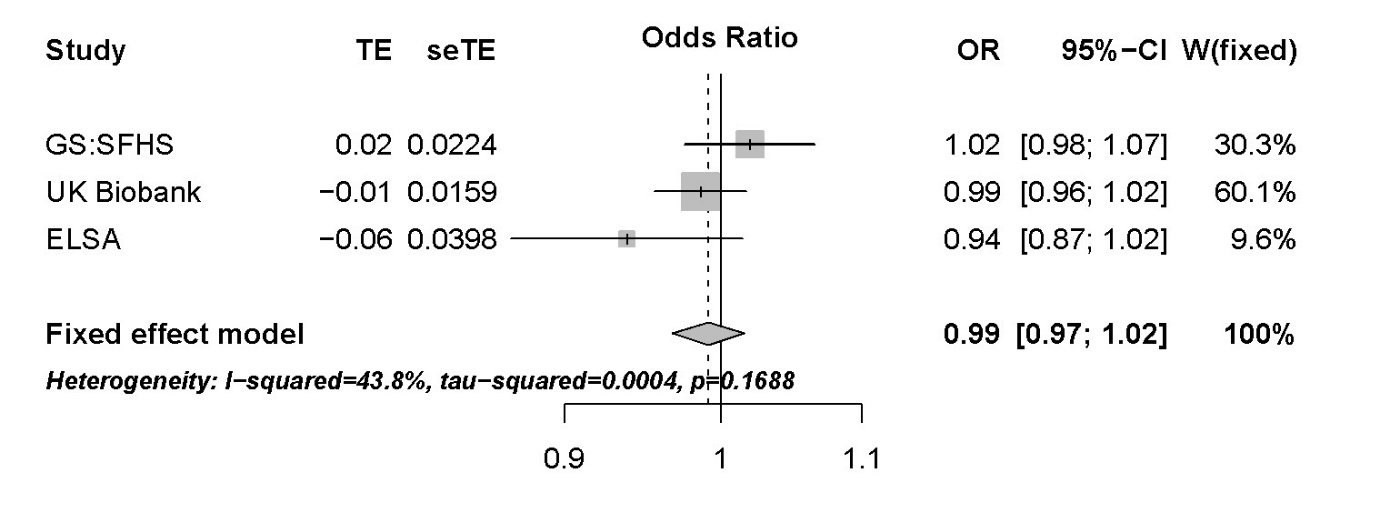
**

**d) ICV PRS**

**
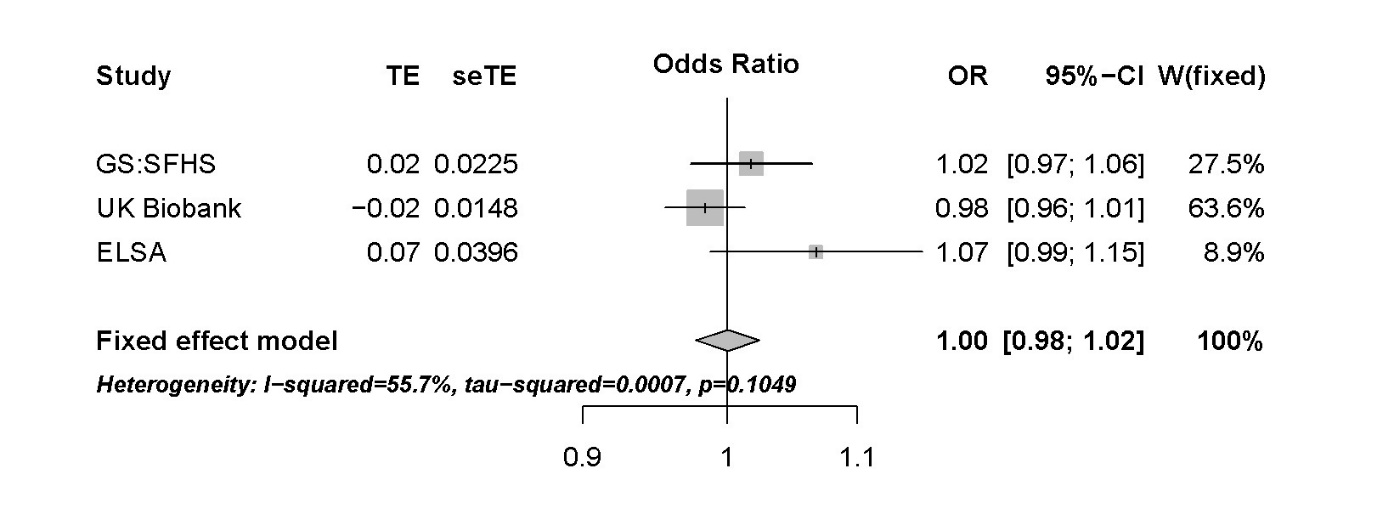
**

**e) Pallidum PRS**

**
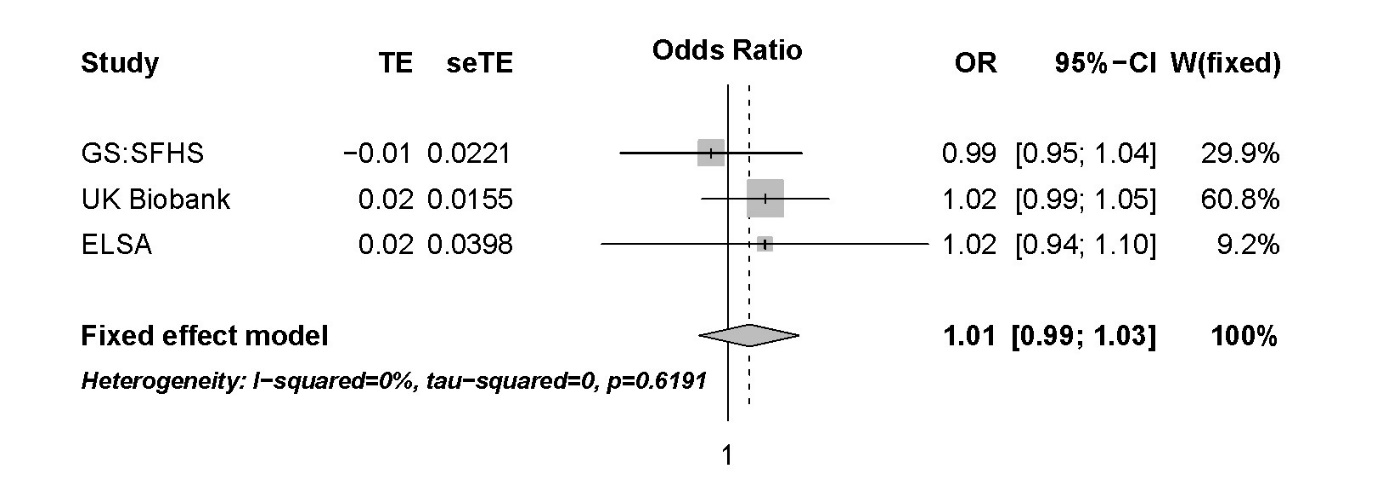
**

**f) Putamen PRS**

**
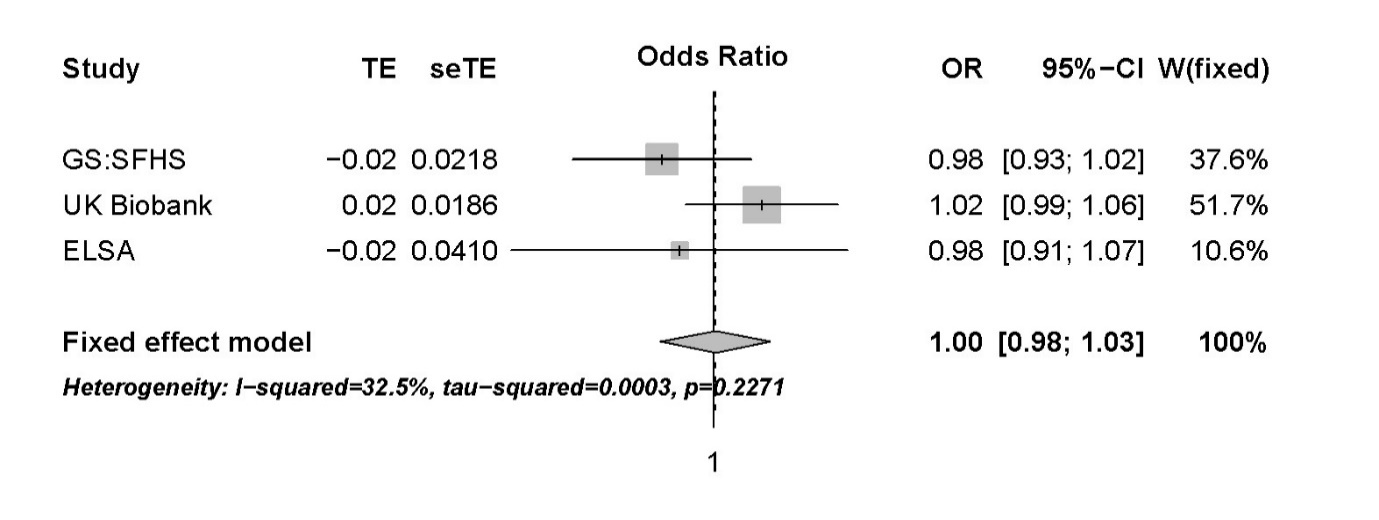
**

**g) Thalamus PRS**

**
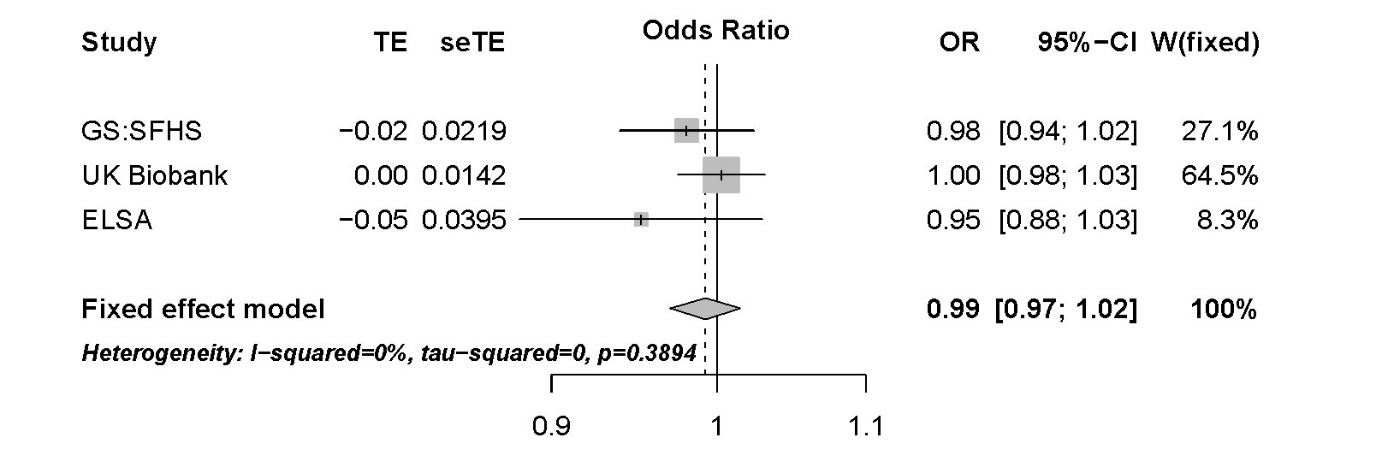
**

No heterogeneity was reported between cohorts but no regional brain PRS is significantly associated with MDD. TE; treatment effect (regression beta’s); seTE, standard errors; OR, odds ratio; CI, confidence intervals; W(fixed), weight of individual studies in fixed effect meta-analysis.

**Figure S5. Meta-analysis forest plots of mixed model analysis of hippocampal volume PRS and MDD episodes, MDD duration and age of onset in GS:SFHS and UK Biobank.**

**S4. a)
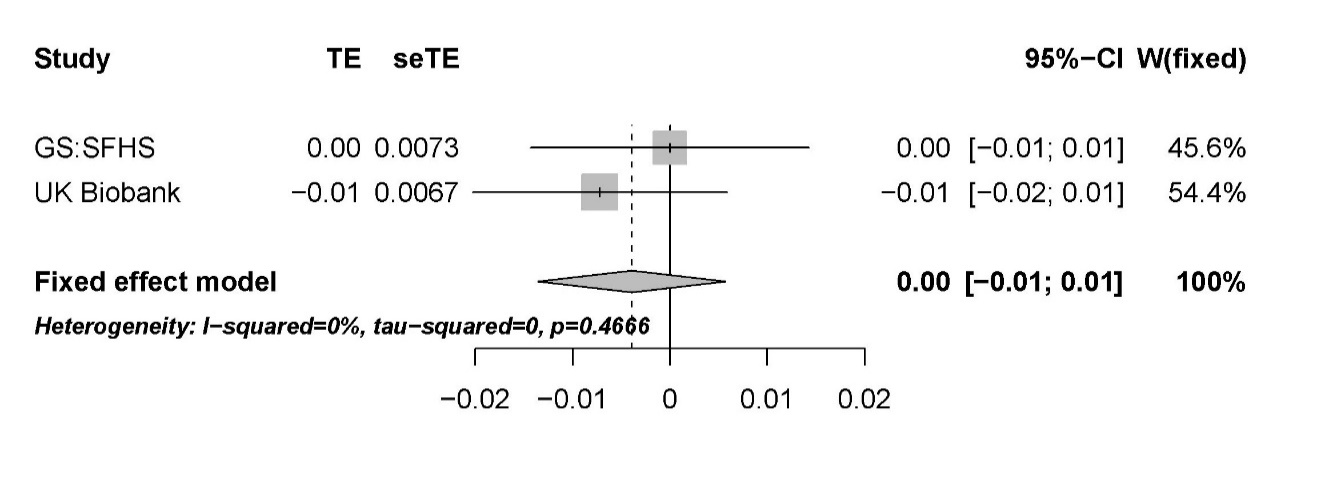
**

**MDD Episodes**

**b)
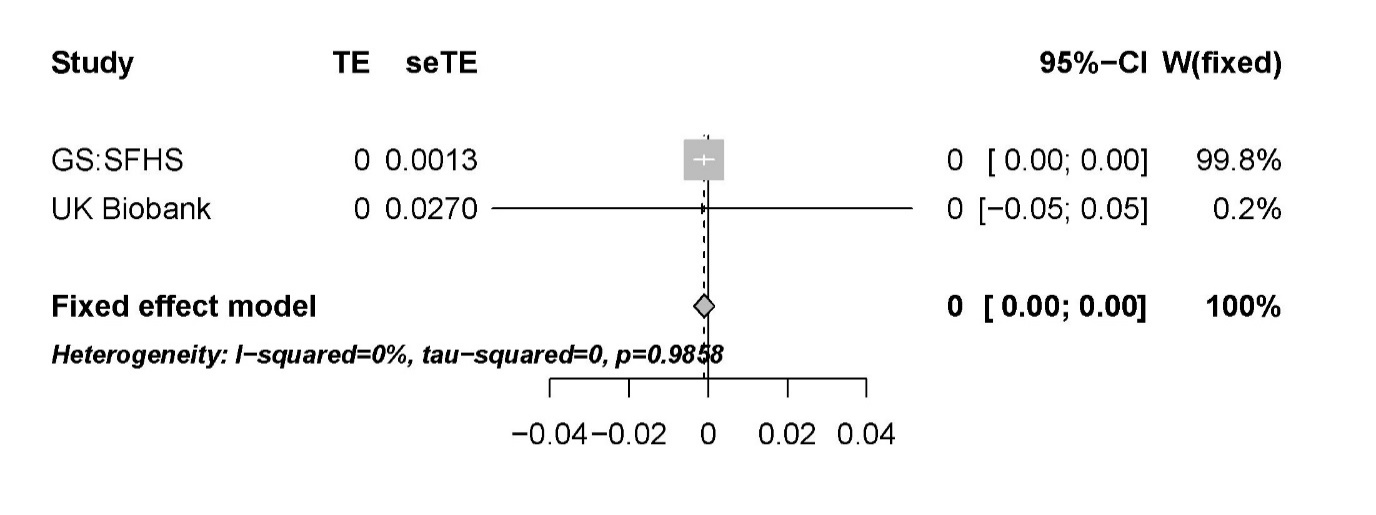
**

**MDD Duration**

**c)
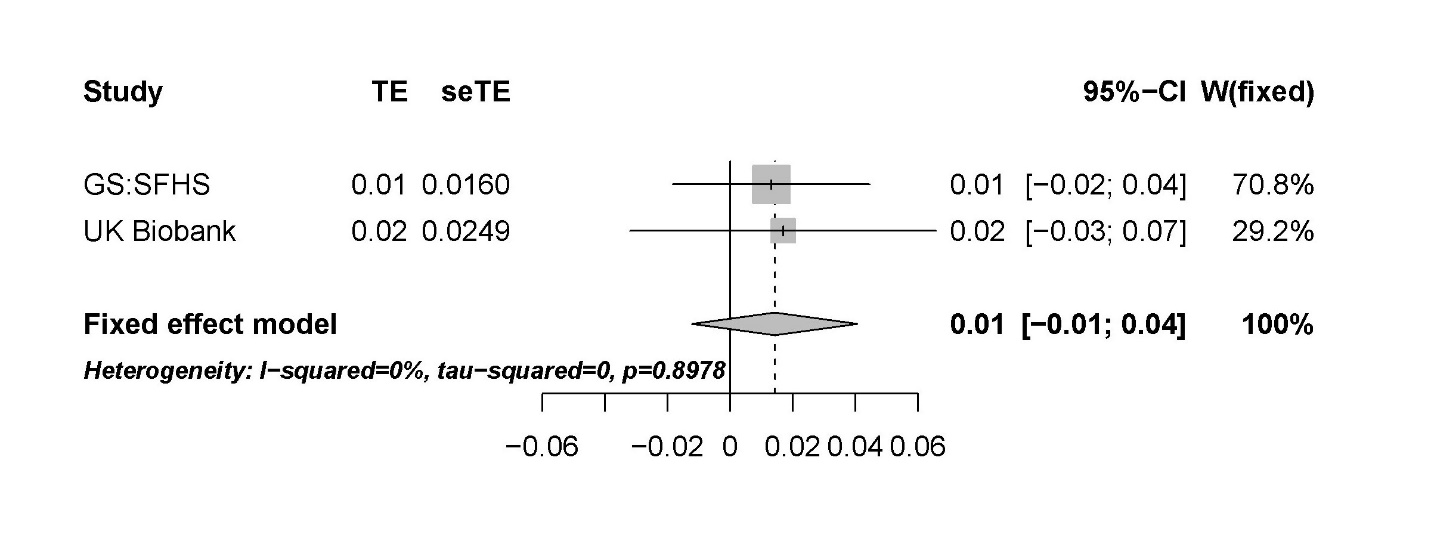
**

**Age of Onset**

No heterogeneity was reported between cohorts but hippocampal volume PRS was not significantly associated with number of MDD episodes (a), MDD duration (b) or age of onset (c). TE; treatment effect (regression beta’s); seTE, standard errors; CI, confidence intervals; W(fixed),weight of individual studies in fixed effect meta-analysis.
